# Processing single-cell RNA-seq datasets using SingCellaR

**DOI:** 10.1016/j.xpro.2022.101266

**Published:** 2022-04-01

**Authors:** Guanlin Wang, Wei Xiong Wen, Adam J. Mead, Anindita Roy, Bethan Psaila, Supat Thongjuea

**Affiliations:** 1 MRC Molecular Haematology Unit, MRC WIMM, University of Oxford, Oxford OX3 9DS, UK; 2 Centre for Computational Biology, Medical Research Council Weatherall Institute of Molecular Medicine (MRC WIMM), University of Oxford, Oxford OX3 9DS, UK; 3 Department of Paediatrics, Children’s Hospital, John Radcliffe Hospital, and MRC WIMM, University of Oxford, Oxford OX3 9DS, UK; 4 National Institute for Health Research (NIHR) Oxford Biomedical Research Centre, Oxford OX4 2PG, UK

**Keywords:** Bioinformatics, Single Cell, RNAseq, Stem Cells, Systems biology

## Abstract

Single-cell RNA sequencing has led to unprecedented levels of data complexity. Although several computational platforms are available, performing data analyses for multiple datasets remains a significant challenge. Here, we provide a comprehensive analytical protocol to interrogate multiple datasets on SingCellaR, an analysis package in R. This tool can be applied to general single-cell transcriptome analyses. We demonstrate steps for data analyses and visualization using bespoke pipelines, in conjunction with existing analysis tools to study human hematopoietic stem and progenitor cells.

For complete details on the use and execution of this protocol, please refer to Roy et al. (2021).

## Highlights

• SingCellaR is an open-source analysis tool for single-cell RNA sequencing data
• SingCellaR facilitates various analyses, including data integration and comparison
• Step-by-step analysis and visualization using SingCellaR for human hematopoietic cells

## Graphical abstract

**Figure:**
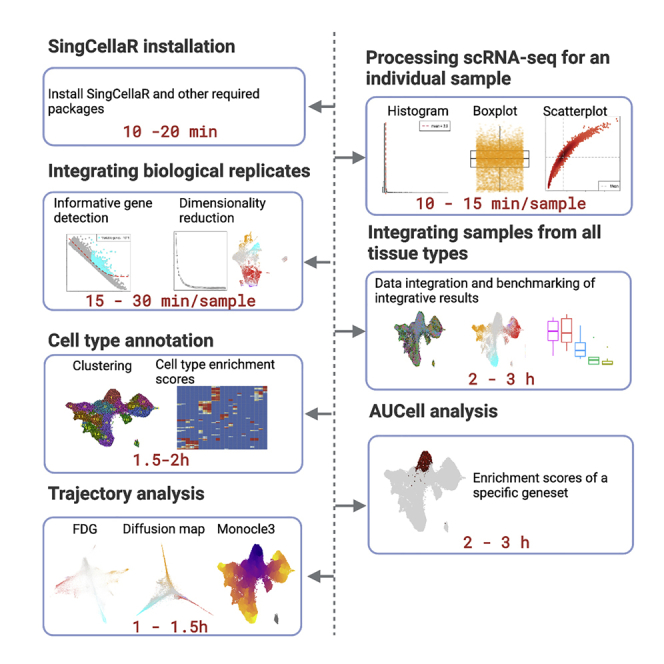

## Before you begin

This protocol describes a method for analyzing single-cell RNA sequencing (scRNA-seq) datasets using an R package called SingCellaR. In addition to standard functions (e.g., reading gene expression matrices, data filtering, doublet removal, dimensionality reduction, data integration, clustering and marker gene identification, and differential gene expression analysis), similar to those performed by available R and Python analysis tools like Seurat (Hao et al., 2021; Stuart et al., 2019), Monocle (Trapnell et al., 2014), and Scanpy (Wolf et al., 2018), SingCellaR supports multiple data integration methods within a single platform. It also incorporates objective benchmarking of the integrative results, facilitating the comparison and choice of the most appropriate integration. To complement existing integration packages, SingCellaR includes a novel integration method - Supervised Harmony (Roy et al., 2021), which showed a better integrative result than available methods for integrating multiple scRNA-seq datasets generated from different hematopoietic tissues.

SingCellaR supports multiple modalities for visualization, including tSNE, UMAP, force-directed graph (FDG) in two- or three-dimensional embeddings, diffusion maps, violin and bubble plots, and heatmaps. Cells can be identified on these plots according to user-defined parameters, such as cluster, donor, tissue of origin, disease state, etc., by accessing relevant information from the SingCellaR object. Multiple signature gene scores can also be superimposed on the same embeddings. For example, visualization of sets of canonical genes used to distinguish different blood cell lineages can help exploration of the cell types contained within a dataset independently of cell clustering algorithms. To annotate cell types and states, SingCellaR uses gene set enrichment analysis to calculate enrichment scores for user-defined, curated gene sets. This system can be used alongside manual inspection for canonical marker genes, or other existing algorithms (Aran et al., 2019; Pliner et al., 2019; Shao et al., 2020; Zhang et al., 2019).

We recently demonstrated the utility of the SingCellaR pipeline in a study of human hematopoiesis. Studying the site- and stage-specific changes of normal hematopoiesis over human development is crucial to understanding the origin of disorders that tend to emerge at specific ages. We analyzed scRNA-seq datasets from hematopoietic stem and progenitor cells (HSPCs) from five different tissues sampled across four stages of the human lifetime (Roy et al., 2021). This study aimed to explore dynamic changes in the cellular compositions of lineage-specific HSPCs and identify the site and developmental stage-specific transitions in gene regulatory networks across human developmental stages.

Here we present a comprehensive protocol for the analytical pipeline demonstrating step-by-step data analysis and visualization as employed in the recent publication (Roy et al., 2021). We cover the software installation, processing of an individual sample and integrating multiple samples with benchmarking of multiple integration methods, cell clustering, cell type annotation, implementation of AUCell, differential abundance, and trajectory analyses. We also provide R functions for single-cell data visualization useful for interpreting results. Finally, limitations, problems, and potential solutions are discussed. All processed datasets, R objects, and codes are available and maintained on the published data repositories.

### scRNA-seq datasets

**Timing: 5–10 min**

In this protocol, we use scRNA-seq datasets of hematopoietic stem and progenitor cells (HSPCs) from human tissues across different developmental stages, including early fetal liver (eFL), matched fetal liver (FL) and bone marrow (FBM) isolated from the same fetuses, pediatric bone marrow (PBM), and adult bone marrow (ABM). Here, human fetal liver samples from first and second trimester are referred to eFL and FL respectively. Table S1 in (Roy et al., 2021) shows the summary of samples and single-cell quality information. Sample collection, fluorescence-activated cell sorting (FACS) strategies, high-throughput sequencing using 10× Genomics platform and data pre-processing steps are described in the method section of (Roy et al., 2021).

We have provided pre-processed datasets and available gene sets on Zenodo: https://doi.org/10.5281/zenodo.5879071. Zenodo (https://zenodo.org/) is an open and citable repository for sharing curation and publication of data and software from research outputs, regardless of data format, size, and access restrictions or license.

The compiled datasets consist of:

1. cellranger pipeline outputs of samples from different tissues: the file ‘cellranger_output.zip’ is a zipped folder containing the cellranger pipeline results of 9 samples (eFL, n= 2; FL, n= 2; FBM, n= 2; PBM, n= 2; ABM, n=1);
2. human signature gene sets that are used in this protocol;
3. codes (Code.zip) used in this protocol;
4. generated R objects from this protocol.

#### Cell Ranger software

(https://support.10xgenomics.com/single-cell-gene-expression/software/pipelines/latest/what-is-cell-ranger) contains analysis pipelines used to align sequencing reads to the reference genome, generate feature-barcode matrices, and perform other downstream analyses. We performed cellranger count as described in https://support.10xgenomics.com/single-cell-gene-expression/software/pipelines/latest/using/count to obtain feature-barcode matrices for each library. The outputs of the pipeline include a Matrix Market file of gene expression (matrix.mtx.gz), cell barcode (barcodes.tsv.gz) and gene metadata (features.tsv.gz) files.

## Key resources table

**Table:** 

REAGENT or RESOURCE	SOURCE	IDENTIFIER
**Deposited data**		

cellranger pipeline outputs pre-processed illustrated in this protocol	(Roy et al., 2021)	https://doi.org/10.5281/zenodo.5879071 https://support.10xgenomics.com/single-cell-gene-expression/software/pipelines/latest/what-is-cell-ranger

**Software and algorithms**		

R (v4.0.4)	Team R C. R: A language and environment for statistical computing[J]. 2013.	https://cran.r-project.org/bin/macosx/ (macOS) https://cran.r-project.org/bin/windows/base/ (Windows) https://cran.r-project.org/bin/linux/ (Linux)
RStudio (v1.1.463)	Team R S. Rstudio: integrated development for R[J]. Rstudio, Inc., Boston, MA URL http://www.Rstudio.com, 2015, 42: 14.	https://www.rstudio.com/products/rstudio/download/
SingCellaR (v1.2.0)	(Roy et al., 2021)	Github: https://github.com/supatt-lab/SingCellaR Zenodo: https://doi.org/10.5281/zenodo.5153387
monocle (v3_0.2.3.0)	(Trapnell et al., 2014)	https://cole-trapnell-lab.github.io/monocle3/
AUCell (v1.14.0)	(Aibar et al., 2017)	https://www.bioconductor.org/packages/release/bioc/vignettes/AUCell/inst/doc/AUCell.html
harmony (v0.1.0)	(Korsunsky et al., 2019)	https://github.com/immunogenomics/harmony
fgsea (v1.18.0)	(Korotkevich et al., 2021)	https://bioconductor.org/packages/release/bioc/html/fgsea.html
DAseq (v1.0.0)	(Zhao et al., 2021)	https://github.com/KlugerLab/DAseq

**Other**		

Computing Platform: • A desktop with local memory 16 GB; 32 GB or higher memory is recommended for large datasets integration. This protocol was performed on macOS Mojave (v10.14.5) with 3.8 GHz Intel Core i5 processor and 32 GB memory. • A high-performance workstation or computing cluster – 32 GB memory or higher with 8-core processor or higher processing cores is recommended.	MacOS Linux	https://www.apple.com https://www.linux.org/

## Step-by-step method details

### SingCellaR installation

**Timing: 10–20 min**

Before we begin, the user has to install SingCellaR and other dependency packages. R packages are hosted across multiple repositories, namely Comprehensive R Archive Network (CRAN), Bioconductor, and Github. For a brief introduction, CRAN is the R central software repository for the latest and previous versions of the R distribution and packages. Bioconductor is the R repository to facilitate R packages developed for biological data analysis. GitHub is a commercial repository that hosts services for individuals and teams for software version control and collaboration. The R functions ‘install.packages’, ‘BiocManager::install’, and ‘devtools::install_github’ will be used to install packages from CRAN, Bioconductor, and Github, respectively. Prior to installation, the function ‘if(!require("package_name"))’ will be used to check if the package has already been installed on a computer, and installation will proceed for packages not yet installed on the computer.

1. **Install SingCellaR from GitHub by running the following R code**:

> if(!require(devtools)) {

> install.packages("devtools")

> }

> if(!require(BiocManager)) {

> install.packages("BiocManager")

> }

> devtools::install_github('supatt-lab/SingCellaR',ref='master',

repos=BiocManager::repositories())

**CRITICAL:** We tested SingCellaR installation on macOS Mojave and Catalina, and Windows 10.

R version 4 or higher is required with other R packages, as shown in the key resources table. SingCellaR incorporates functions from Python (version 3.8) modules that have to be installed as described below. The timing of this step depends on the installation of devtools and Bioconductor packages. Devtools package is an R package to support the installation for other packages that are not yet in a standard package repository, such as CRAN or Bioconductor. R packages, including SingCellaR, usually depend on other R packages (dependencies) to support their full range of functionalities. Therefore, the time to complete the installation depends on the number of dependencies that have to be installed on the computer.

2. Install required python modules by running the following R code:
  a. Modules required for the force-directed graph analysis.
    > library(reticulate)
    > conda_create("r-reticulate", python_version="3.8")
    > py_install("fa2", envname="r-reticulate")
    > py_install("networkx", envname="r-reticulate")
  b. Module required for doublet removal using Scrublet.
    > py_install("Scrublet",envname="r-reticulate")
    **CRITICAL:** We tested the fa2 python module for the force-directed graph analysis using Python versions 2.7, 3.6 and 3.8. Using Conda environment is recommended in conjunction with reticulate package. Conda (https://docs.conda.io/en/latest/) is a virtual environment management system for Python. With Conda, the user can create, remove, and update environments that have different versions of Python packages installed in them. This flexibility is especially helpful on devices in which the user does not have administrative privileges to install Python packages on the device and compile version control for specific packages.

3. Install required R packages by running the following R code:
  a. harmony – required for data integration using Harmony method (Korsunsky et al., 2019).
    > if(!require(harmony)) {
    > install.packages("harmony")
    > }
  b. AUCell – required for computing AUCell scores with specified gene signatures (Aibar et al., 2017).
    > if(!require(AUCell)) {
    > BiocManager::install("AUCell")
    > }
  c. doParallel and doRNG – required for parallel processing in AUCell analysis.
    > if(!require(doParallel)) {
    > install.packages("doParallel")
    > }
    > if(!require(doRNG)) {
    > install.packages("doRNG")
    > }
  d. DAseq – required for the analysis of differential abundance (Zhao et al., 2021).
    > if(!require(DAseq)) {
    > devtools::install_github("KlugerLab/DAseq")
    > }
  e. destiny – required for the trajectory analysis using diffusion map (Haghverdi et al., 2015).
    > if(!require(destiny)) {
    > BiocManager::install("destiny")}
  f. monocle3 – required for the trajectory analysis (Trapnell et al., 2014).
    > devtools::install_github('cole-trapnell-lab/monocle3',
    ref="develop")
    ***Note:*** The destiny package is not available for Bioconductor version 3.13. The user can install this package from GitHub.
    > install_github("https://github.com/theislab/destiny",
    build_vignettes=FALSE, dependencies=TRUE)
    ***Optional:*** SingCellaR supports multiple dataset integration and batch correction methods including Scanorama (Hie et al., 2019), Seurat Canonical Correlation Analysis (CCA) and Reciprocal PCA (RPCA) (Hao et al., 2021), Liger with online integrative non-negative matrix factorization (iNMF) (Gao et al., 2021), ComBat (Johnson et al., 2007) and Limma (Ritchie et al., 2015).
  g. scanorama – required for the data integration.
    The user can install scanorama using pip command line:
    > pip install scanorama
  h. Seurat CCA/RPCA – required for the data integration.
    > install.packages("Seurat")
  i. rliger – required for the data integration.
    > install.packages("rliger")
  j. ComBat – required for the batch removal.
    > if (!requireNamespace("BiocManager", quietly = TRUE))
     install.packages("BiocManager")
    > BiocManager::install("sva")
  k. Limma – required for the batch removal.
    > if (!requireNamespace("BiocManager", quietly = TRUE))
     install.packages("BiocManager")
    > BiocManager::install("limma")

### Processing scRNA-seq for an individual sample

**Timing: 10–15 min for each sample, depending on the number of cells being analyzed**

This step creates a SingCellaR object for each sample and performs quality control (QC) to identify cells that qualify for further downstream analyses. We demonstrate the selection process of high-quality cells using multiple QC plots, data normalization, and identifying highly variable genes. The process starts from reading in the input files for each sample generated directly from the cellranger pipeline. Here, we show an early fetal liver (eFL_1) dataset as an example.

4. Load SingCellaR package into R environment:

> library(SingCellaR)

> setwd("./SingCellaR_objects")

***Note:*** ‘./SingCellaR_objects’ is a local folder for this analysis. The user can change the folder name to a suitable file path in a local computer or server.

5. **Create SingCellaR object.** SingCellaR object is an extension of the SingleCellExperiment (Amezquita et al., 2020) object for storing data generated from single-cell experiments. The R code below shows how to read in the input files for generating the SingCellaR object using the function ‘load_matrices_from_cellranger’. The input files are from the output of the cellranger pipeline version 3.0.1. The folder eFL1 contains input files consisting of cell barcodes (barcodes.tsv.gz), gene features (features.tsv.gz), and matrix (matrix.mtx.gz) containing unique molecular identifier (UMI) counts for gene expression. The user can assign a unique identifier to the sample in this step.

> data_matrices_dir<-"./cellranger_output/eFL1/"

> eFL_1<-new("SingCellaR")

> eFL_1@dir_path_10x_matrix<-data_matrices_dir

> eFL_1@sample_uniq_id<-"eFL_1"

> load_matrices_from_cellranger(eFL_1,cellranger.version = 3)

> eFL_1

Now the SingCellaR object – eFL_1 is created.

**CRITICAL:** The ‘cellranger.version’ parameter is required to be compatible with the cellranger pipeline output file. The cellranger output file ‘features.tsv.gz’ for gene features is generated from Cell Ranger version 3 and above, whereas version 2 of Cell Ranger creates the file ‘genes.tsv.gz’.

**Figure 1:**
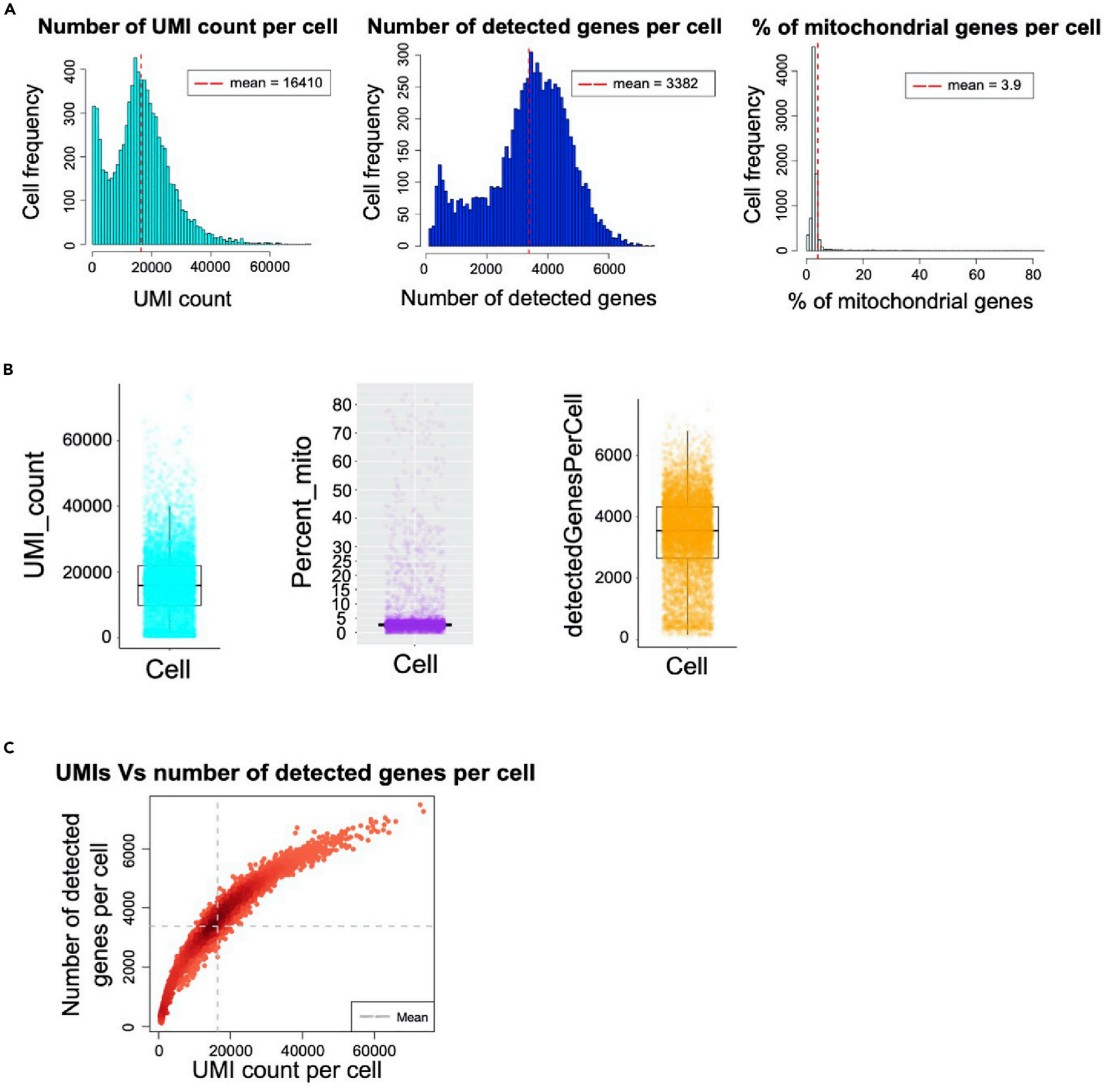
SingCellaR visualization of QC matrices for a single sample (A) Histograms show the cell frequency based on the number of UMI counts per cell, the number of detected genes per cell, and the percentage of mitochondrial gene expression per cell. Dashed lines represent the mean. (B) The same QC matrices as in (A) but represented using boxplots that show the distribution of cells (dots). (C) Scatterplot showing the number of UMI counts per cell (x-axis) and the number of detected genes per cell (y-axis); These panels can be used to determine the cell filtering criteria for selecting high-quality cells. Dashed lines represent the mean of detected genes and UMI counts per cell.

6. **Create cell metadata.** Cell metadata will be created. Rows represent cells and columns represent variables. Variables computed in this step include the number of UMIs and detected genes per cell, and percentage of mitochondrial gene expression for each cell.
  > process_cells_annotation(eFL_1, mito_genes_start_with="MT-")
  The following parameter is required:
  a. **mito_genes_start_with:** Gene names starting with ‘MT-’ are used as a set of mitochondrial genes for human and ‘mt-’ for mouse samples.
    ***Note:*** The cell metadata can be accessed using the function ‘get_cells_annotation(eFL_1)’ or eFL_1@meta.data. The user can manually add additional information to the columns of the cell metadata.

7. **Visualize QC matrices.** QC matrices computed in step 6 can be explored using the plotting functions:
  a. Histogram (Figure 1A).
    > plot_cells_annotation(eFL_1,type="histogram")
  b. Boxplot (Figure 1B).
    > plot_cells_annotation(eFL_1,type="boxplot")
  c. Plot of the number of UMIs versus the number of detected genes per cell (Figure 1C).
    > plot_UMIs_vs_Detected_genes(eFL_1)

8. **Annotate cell quality, identify expressed genes, and assign cell and gene status into metadata.** After visualizing QC matrices in step 7, the user can specify filtering parameters using the observed number of UMIs and detected genes per cell, and percentage of mitochondrial gene expression. The function ‘filter_cells_and_genes’ assigns a new column named IsPassed that will be added into the cell metadata. Cells that pass QC will be annotated as TRUE. Expressed genes will be identified in this step and the column named IsExpress will be added into the gene metadata. Genes expressed above and below the user-defined threshold will be annotated as TRUE or FALSE. Although all original cells and genes are retained in the metadata and gene expression matrix in this step, these annotations will be used to subset cells and expressed genes for further downstream analyses. The number of cells passing QC thresholds will be shown on the R console after running the following code:
  > filter_cells_and_genes(eFL_1,
     min_UMIs=1000,
     max_UMIs=50000,
     min_detected_genes=500,
     max_detected_genes=6000,
     max_percent_mito=10,
     genes_with_expressing_cells = 10,
     isRemovedDoublets = FALSE)
  The following parameters are described:
  a. **min_UMIs:** The lower threshold for UMI counts, above which cells are annotated as high-quality. To be used in conjunction with max_UMIs argument. Default value is 1,000.
  b. **max_UMIs:** The upper threshold for UMI counts, below which cells are annotated as high-quality. To be used in conjunction with min_UMIs argument. Default value is 30,000.
  c. **min_detected_genes:** The lower threshold for the number of expressed genes, above which cells are annotated as high-quality. To be used in conjunction with max_detected_genes argument. Default value is 1,000.
  d. **max_detected_genes:** The upper threshold for the number of expressed genes, below which cells are annotated as high-quality. To be used in conjunction with min_detected_genes argument. Default value is 8,000.
  e. **genes_with_expressing_cells:** The lower threshold for the number of cells in which a gene is expressed (UMI >=1), above which, the gene will be annotated as expressed. Default value is 10.
  f. **isRemovedDoublets:** If set to TRUE (default), doublets will be removed prior to downstream analyses.
    ***Note:*** Parameters used for filtering out cells with low quality can be varied across tissues and organs from different datasets. For example, the percentage threshold for mitochondrial gene expression, ‘max_percent_mito’ can be set higher for liver tissues (MacParland et al., 2018). The user can use the plotting functions as described above to explore different cut-offs for all samples.
    ***Optional:*** In addition, we have incorporated the Scrublet package (Wolock et al., 2019) for doublet removal into the latest version of SingCellaR. The user can add the optional step below to detect and exclude doublets:
    > DoubletDetection_with_scrublet(eFL_1)

9. **Normalize UMI counts.** SingCellaR scales UMI counts by normalizing each library size to 10,000 or mean library size.

> normalize_UMIs(eFL_1, use.scaled.factor = T)

The following parameter is required:

**use.scaled.factor:** When set to TRUE, the gene expression values will be multiplied by 10,000 (by default) and normalized against the library size (total UMI counts) of each cell. The user can change the scale factor value using the scale.factor parameter. If set to FALSE, the function will use the mean of library size across all cells as the scale factor.

**CRITICAL:** Using use.scaled.factor=TRUE is recommended for 10× genomics data. The user should consider specifying use.scaled.factor=FALSE for scRNA-seq data generated from plate-based protocols, e.g., Smart-seq2 (Picelli et al., 2014) and TARGET-Seq (Rodriguez-Meira et al., 2019). This is because the sequencing depth of scRNA-seq data generated from the plate-based protocols is in the higher ranges compared to 10× genomics data (e.g., from a hundred thousand to millions of reads per cell). Thus, using a scale factor of 10,000 is insufficient and leads to small gene expression values. The user should set use.scaled.factor=FALSE in order to use the mean library size from all single cells as the scale factor.

10. **Identify highly variable genes.** SingCellaR uses a gamma generalized linear model (GLM) as the fitting method for gene expression and coefficient of variation to identify highly variable genes. The GLM is suitable for modeling log-normal data from a sparse normalized gene expression matrix. A column named IsVarGenes is added to the gene metadata and genes identified as highly variable are annotated as TRUE, while all other genes are annotated as FALSE. The number of genes used to fit in the model and the number of identified variable genes will be shown after running the following code:
  > get_variable_genes_by_fitting_GLM_model(eFL_1,
     mean_expr_cutoff = 0.05,
     disp_zscore_cutoff = 0.05)
  The following parameters are required:
  a. **mean_expr_cutoff:** The mean normalized expression value, above which, the genes are identified as highly variable. Default value is 0.1.
  b. **disp_zscore_cutoff:** The dispersion of z-score, above which, the genes are identified as highly variable. Default value is 0.1.
    ***Note:*** In this step, we used lower cut-off values to increase the number of detected highly variable genes per sample for the downstream analyses.

11. Save the R object for further analyses.

> save(eFL_1, file="./eFL_1.SingCellaR.rdata")

12. **Repeat the analyses for the rest of samples.** R codes are available at Zenodo: https://doi.org/10.5281/zenodo.5879071.

**Pause point:** The user can pause the analysis after pre-processing each sample.

### Integrating biological replicates

**Timing: 15–30 min for each group, 1–2 h for all stages**

This step integrates the individual R objects from pre-processed biological or technical replicates generated from step 12. Here, we illustrate the integration of two early fetal liver samples collected from two donors (Roy et al., 2021).

13. Load SingCellaR package.

> library(SingCellaR)

14. **Integrate pre-processed biological replicates.** The user will initialize the integrated SingCellaR class object ‘SingCellaR_Int’ and assign a unique identifier prior to merging two datasets using the ‘preprocess_integration’ function.

> eFL <- new("SingCellaR_Int")

> eFL@dir_path_SingCellR_object_files<-"./"

> eFL@SingCellR_object_files=c("eFL_1.SingCellaR.rdata",

     "eFL_2.SingCellaR.rdata")

> preprocess_integration(eFL)

> eFL

15. **Annotate cell quality and expressed genes.** Filtering process has been already performed separately for each sample (see step 8), therefore the filtering parameters for this step will be set to include all cells. From the filtering output below, all cells will be retained, after running the following code:

> filter_cells_and_genes(eFL,

    min_UMIs=1000,

    max_UMIs=50000,

    min_detected_genes=500,

    max_detected_genes=6000,

    max_percent_mito=10,

    isRemovedDoublets = FALSE)

16. **Normalize and scale UMI counts.**

> normalize_UMIs(eFL, use.scaled.factor = T)

***Note:*** See step 9 for details of required parameters.

17. **Regress out confounding factors.** The normalized and scaled gene expression values from step 16 will be adjusted by regressing out cell-to-cell variation in gene expression values due to confounding factors (e.g., batch and donor effect). To this end, SingCellaR implements the ‘lmFit’ function from the R package limma (Ritchie et al., 2015), SingCellaR provides the ‘remove_unwanted_confounders’ wrapper function to regress out the unwanted source of variation. Here, we will regress out the effects of UMI counts, percentage of mitochondrial gene expression, and donor. Adjusted gene expression will be used for further analyses.
  > remove_unwanted_confounders(eFL,
   residualModelFormulaStr="∼UMI_count+percent_mito+sampleID")
  The following parameter is required:
  a. **residualModelFormulaStr:** The formula format used to regress out confounding factors. The names of variables defined have to be the same as the column names of the cell metadata.
    ***Note:*** The user can change the residualModelFormulaStr parameter and perform the following steps down to step 23 (UMAP analysis) to explore the effect on cell clustering by specifying different sets of confounding factors. For example, the user can set residualModelFormulaStr = "∼UMI_count+percent_mito" to compare with residualModelFormulaStr = "∼UMI_count+percent_mito+sampleID" to explore the effect of sample.

18. **Identify highly variable genes.** The number of genes used for fitting the GLM model and the number of highly variable genes will be identified after running the following code:

> get_variable_genes_by_fitting_GLM_model(eFL,

     mean_expr_cutoff = 0.05,

     disp_zscore_cutoff = 0.05)

***Note:*** See step 10 for details of required parameters.

19. **Remove selected genes.** Here, we remove mitochondrial and ribosomal genes from highly variable genes identified from step 18 to avoid the skewed effect of ribosomal and mitochondrial gene expression in downstream analyses. The number of genes that are excluded will be shown after running the following code:

> remove_unwanted_genes_from_variable_gene_set(eFL,

 gmt.file = "./Human_genesets/human.ribosomal-mitochondrial.genes.gmt",

 removed_gene_sets=c("Ribosomal_gene","Mitochondrial_gene"))

***Note:*** The human.ribosomal-mitochondrial.genes.gmt file can be downloaded from Zenodo: https://doi.org/10.5281/zenodo.5879071 under the folder Human_genesets.

**Figure 2:**
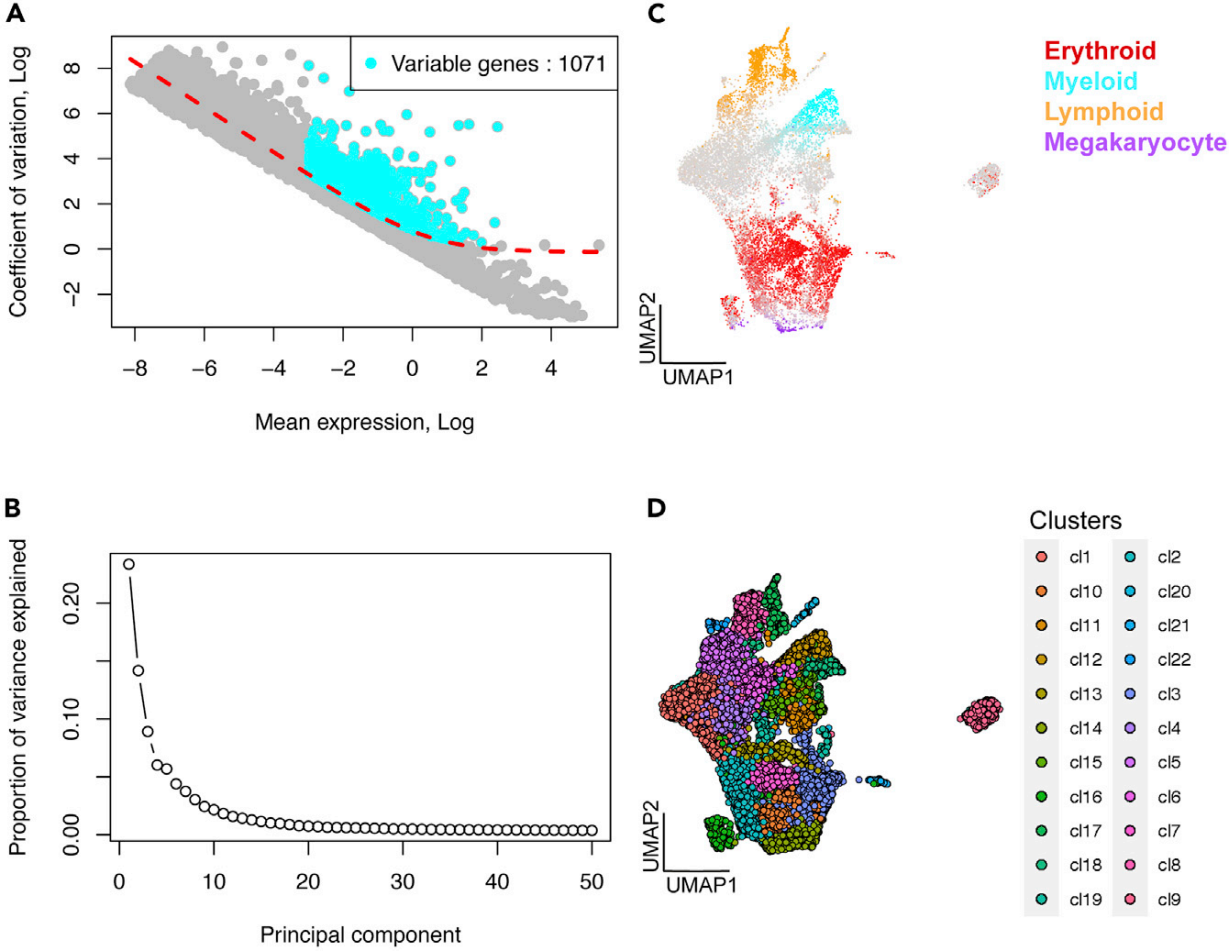
Visualization of highly variable genes and UMAP analysis results (A) Highly variable genes are identified and shown on the fitted gamma generalized linear model (GLM) plot. Gray dots represent all genes that are used to fit in GLM model. Light blue dots represent genes identified as highly variable genes that pass the gene expression cutoff and are fitted above the general fitted lines (red dashed-line). (B) The elbow plot represents the proportion of variance (y-axis) captured by the principal component analysis (PCA) and are ranked from PC1 to PC50 (x-axis). This plot can be used to determine the number of PCs to include in the downstream analyses; The first 30 PCs are used in this case. (C) The UMAP plot shows the expression of lineage gene sets. Yellow – lymphoid cells; Cyan – myeloid cells; Red – erythroid cells; Purple – megakaryocytic cells; Gray – HSPCs that do not (or lowly) express lineage signature genes. (D) The UMAP plot shows identified Louvain clusters.

20. Visualize highly variable genes (Figure 2A).

> plot_variable_genes(eFL)

21. **Perform principal component analysis (PCA).** To interpret the relationship across single cells, dimensionality reduction methods are required to reduce high dimensionality data to the visualizable two- or three-dimensional space. In PCA, the reduced dimensional space is represented by principal components. The top PCs will capture most of the variance of the dataset. Here, we perform linear dimensionality reduction using PCA and then bring forward the most informative PCs for further nonlinear dimensionality reduction to visualize cells in a two-dimensional space. To this end, highly variable genes identified from steps 18–19 and visualized in step 20 will be used for PCA using the ‘runPCA’ function, a wrapper function for the ‘irlba’ function from the irlba package (http://bwlewis.github.io/irlba/).
  > SingCellaR::runPCA(eFL, use.components=50, use.regressout.data = T)
  The following parameters are required:
  a. **use.components:** The number of principal components (PCs) to estimate. Default value is 50.
  b. **use.regressout.data:** If set to TRUE (default), the adjusted gene expression values from step 17 will be used.

22. **Visualize principal components.** The elbow plot is used to determine the number of PCs to be included for further dimensionality reduction analyses (Figure 2B). The number of PCs to specify downstream should correspond to the elbow point, used as a cut-off to include PCs that capture most of the biological variations found in the data.

> plot_PCA_Elbowplot(eFL)

23. **Perform nonlinear dimensionality reduction analyses.** Running nonlinear dimension reduction on all highly variable genes requires high computational resources and processing time. Hence, using identified PCs from step 22 for nonlinear dimension reduction analysis is a standard technique for scRNA-seq analysis. Nonlinear dimension reduction analysis is suitable for capturing cellular heterogeneity (Xiang et al., 2021). Here, we use Uniform Manifold Approximation and Projection (UMAP) (McInnes et al., 2018) by running the ‘runUMAP’ wrapper function that implements the ‘umap’ function from the uwot package (https://cran.r-project.org/web/packages/uwot/). Based on the elbow plot shown in step 22, we select the first 30 PCs for UMAP analysis.
  > SingCellaR::runUMAP(eFL,
     dim_reduction_method = "pca",
     n.dims.use = 30,
     n.neighbors = 30,
     uwot.metric = "euclidean")
  The following parameters are required:
  a. **dim_reduction_method**: The method name for the linear dimension reduction.
  b. **n.dims.use:** The number of selected PCs as determined in step 22.
  c. **n.neighbors:** The number of neighboring cells (the size of the local neighborhood) used for manifold approximation. Default value is 30.
  d. **uwot.metric:** The distance metric name. Default is ‘cosine’.
    ***Note:*** The user can also apply the t-Distributed Stochastic Neighbor Embedding (tSNE) approach using the ‘runTSNE’ function. The reduced dimension coordinates for UMAP and tSNE can be accessed using functions ‘get_umap.result’ and ‘get_tsne.result’ respectively.

24. **Visualize cell lineages on low dimensional space.** To explore whether the cells in each lineage are clustered in close proximity, we will visualize the UMAP result with the expression of multi-lineage gene sets using the ‘plot_umap_label_by_multiple_gene_sets’ function (Figure 2C). Here, we observe that cells are clustered together according to major cell types.
  > plot_umap_label_by_multiple_gene_sets(eFL,
   gmt.file = "./Human_genesets/human.signature.genes.v1.gmt",
   show_gene_sets = c("Erythroid","Myeloid","Lymphoid","Megakaryocyte"),
   custom_color = c("red","cyan","orange","purple"),
   isNormalizedByHouseKeeping = F,
   point.size = 1)
  The following parameters are required:
  a. **gmt.file:** Path to the file containing the gene signatures in GMT format.
  b. **show_gene_sets:** The names of the gene signatures to plot on UMAP.
  c. **custom_color:** The color assignment to each signature specified in ‘show_gene_sets’.
  d. **isNormalisedByHouseKeeping:** When set to TRUE (default), the gene expression values of the individual genes of each gene signature specified will be normalized by the housekeeping genes. The housekeeping genes are defined as the top 100 genes with the highest total gene expression values across all cells.
  e. **point.size:** Size of the data points on UMAP plot. Default value is 2.
    ***Note:*** Lineage gene sets (human.signature.genes.v1.gmt) are available at Zenodo: https://doi.org/10.5281/zenodo.5879071 under Human_genesets folder and from original publication (Roy et al., 2021).

25. **Detect and assign clusters.** To detect and assign cell clusters, the ‘identifyClusters’ function is used. By default, the Louvain community-detection method implemented in the igraph package (https://igraph.org/r) is used for cell clustering.
  > identifyClusters(eFL,n.dims.use = 30,
     dim_reduction_method = "pca",
     n.neighbors = 30,
     knn.metric = "euclidean")
  The following parameters are required:
  a. **n.dims.use:** The number of PCs to use. Default number is 30.
  b. **dim_reduction_method:** The dimensionality reduction analysis name.
  c. **n.neighbors:** The number of neighboring cells. This number may be the same as specified in step 23 if UMAP is used. Default number is 30.
  d. **knn.metric:** The distance metric, ‘euclidean’ is used by default. Another option is ‘cosine’.
    ***Note:*** The cluster metadata for each cell can be accessed using the ‘get_cluster’ function.

26. **Visualize clusters on low dimensional space.** Louvain clusters can be shown on UMAP using the ‘plot_umap_label_by_clusters’ function (Figure 2D).
  > plot_umap_label_by_clusters(eFL,show_method = "louvain",mark.clusters = F)
  The following parameters are required:
  a. **show_method:** The clustering detection name used as in step 25.
  b. **mark.clusters:** If set to TRUE (default) , cluster identifiers will be shown on the plot.

27. **Identify cluster-specific genes.** The user can identify marker genes, which are particularly expressed in each cluster using the ‘findMarkerGenes’ function. Differentially expressed gene analysis between one cluster against all other clusters is performed using the nonparametric Wilcoxon test on normalized expression values for the comparison of expression level and Fisher’s exact test for the comparison of expressing cell frequency (Giustacchini et al., 2017). *P*-values generated from both tests are then combined using Fisher’s method and are adjusted using Benjamini–Hochberg (BH). This process is iterated for each cluster against all other clusters, therefore the processing time in this step is dependent on the number of cells and clusters. By default, the minimum log2 fold change ‘min.log2FC’ parameter is set to 0.5 and the minimum fraction of expressing cells in each cluster ‘min.expFraction’ parameter is set to 0.3.
  > findMarkerGenes(eFL,cluster.type = "louvain")
  The following parameter is required:
  a. **cluster.type:** The clustering detection method used in step 25.

28. **Save R object for further analyses.**

> save(eFL,file, “./eFL_All.SingCellaR.rdata")

29. **Repeat the integration process for all biological replicates of FL, FBM, and PBM samples.** There is only one sample for ABM. Therefore, data integration is not required for this sample at this step. All R codes provided for each integration are available at Zenodo: https://doi.org/10.5281/zenodo.5879071.

**Pause point:** The user can pause the analysis after integrating the biological replicates for each developmental stage and save the results in multiple SingCellaR objects.

### Integrating samples from all tissue types

**Timing: 2–3 h**

The aim of integrating all samples is to assess the existence of batch or donor-specific effects that are confounding factors contributing to differences in gene expression profile across samples. Examples of batch effect include differences in library preparation methods, sequencing batch, and donor or sample ID (Tran et al., 2020). If the batch effect is observed, it can be adjusted by a variety of existing batch correction and integration methods incorporated in SingCellaR, including a novel technique, namely Supervised Harmony that we have developed and described in (Roy et al., 2021). SingCellaR also implements the wrapper functions for other integration methods including Harmony (Korsunsky et al., 2019), Seurat (Hao et al., 2021), Liger (Gao et al., 2021), Scanorama (Hie et al., 2019), Combat (Johnson et al., 2007), and Limma (Ritchie et al., 2015). In this step, we demonstrate examples of how to use these methods for data integration using the wrapper functions. A strategy to benchmark how well single cells are clustered across covariate variables (e.g., batch and donor) is illustrated.

General examples of data integration include integrating samples from healthy donors and patients (Granja et al., 2019; Psaila et al., 2020) and from patients at different disease stages such as in cancer at diagnosis, remission, and relapse. Here, we illustrate the integration of HSPCs from five tissues spanning four different stages of human development that also happened to be processed and sequenced in two different batches (Roy et al., 2021).

30. Load SingCellaR package.

> library(SingCellaR)

31. Initialize the SingCellaR_Int object and merge datasets generated from step 29.

> Human_HSPC <- new("SingCellaR_Int")

> Human_HSPC@dir_path_SingCellR_object_files<-"./"

> Human_HSPC@SingCellR_object_files=c("eFL_All.SingCellaR.rdata",

     "FL_All.SingCellaR.rdata",

     "FBM_All.SingCellaR.rdata",

     "PBM_All.SingCellaR.rdata",

     "ABM_1.SingCellaR.rdata")

> preprocess_integration(Human_HSPC)

> Human_HSPC

32. **Annotate cell quality.** Input parameters for integrated samples have been set to include all cells. The user should observe that there are no cells being filtered out after running the following code:

> filter_cells_and_genes(Human_HSPC,

   min_UMIs=1000,

   max_UMIs=60000,

   min_detected_genes=500,

   max_detected_genes=6000,

   max_percent_mito=20,

   isRemovedDoublets = F)

33. **Incorporate donor and sequencing batch information into cell metadata.** This information is required to perform the batch correction.

> meta.data <- read.delim(file = "./meta.data.txt", header = T, sep = "∖t")

> Human_HSPC@meta.data<- meta.data

Updated meta.data can be checked by running:

> head(Human_HSPC@meta.data)

34. Normalize and scale UMI counts.

> normalize_UMIs(Human_HSPC, use.scaled.factor = T)

35. Identify highly variable genes.

> get_variable_genes_by_fitting_GLM_model(Human_HSPC,

     mean_expr_cutoff = 0.05,

     disp_zscore_cutoff = 0.05)

36. Remove ribosomal and mitochondrial genes.

> remove_unwanted_genes_from_variable_gene_set(Human_HSPC,

 gmt.file = "./Human_genesets/human.ribosomal-mitochondrial.genes.gmt",

 removed_gene_sets=c("Ribosomal_gene","Mitochondrial_gene"))

37. Visualize highly variable genes.

> plot_variable_genes(Human_HSPC)

38. Run PCA.

> SingCellaR::runPCA(Human_HSPC,

    use.components = 100,

    use.regressout.data = FALSE

    )

39. **Visualize principal components.** Based on the elbow plot, the first 40 PCs will be used for data integration.

> plot_PCA_Elbowplot(Human_HSPC)

40. **Integrate data using Supervised Harmony.** We introduce Supervised Harmony, a method for data integration implemented in SingCellaR. Supervised Harmony can be performed using the ‘runSupervised_Harmony’ function. This method is an adaptation of Harmony method (Korsunsky et al., 2019). More details of this method were described (Roy et al., 2021). Here, sequencing batch and donor IDs as defined in step 33 are specified as covariates.
  > SingCellaR::runSupervised_Harmony(Human_HSPC,
      covariates = c("batch","donor"),
      n.dims.use = 40,
      hcl.height.cutoff = 0.3,
      harmony.max.iter = 20,
      n.seed = 6)
  The following parameters are required:
  a. **covariates:** The name(s) of the covariate(s) specified as batch effect to be adjusted. The names should be the same as the column names of the cell metadata.
  b. **n.dims.use:** The number of PCs as determined from step 39 to be used in this step.
  c. **hcl.height.cutoff:** The cutree cut-off value for hierarchical clustering. Default value is 0.25.
  d. **harmony.max.iter:** The maximum number of rounds to run harmony. Default value is 10.
  e. **n.seed:** The random seed number generator. Default value is 1.
    **CRITICAL:** Before running Supervised Harmony method, the ‘findMarkerGenes’ function must be performed for each developmental stage analysis (see step 27). The seed number (random number generator) and software version can vary across different devices. Hence, the user may notice variations in the rotation of the plots and clusters, which can be verified and visualized using lineage genes (see step 24).

41. Nonlinear dimension reduction analysis.

> SingCellaR::runUMAP(Human_HSPC,

    useIntegrativeEmbeddings = T,

    integrative_method = "supervised_harmony",

    umap_method = "uwot",

    n.dims.use = 40,

    uwot.metric = "euclidean",

    n.seed = 1)

The UMAP analysis result from Supervised Harmony integration will be saved. This UMAP object will be used to compare with the results from other integrative methods (see steps below).

> supervised_harmony.UMAP<-get_umap.result(Human_HSPC)

> saveRDS(supervised_harmony.UMAP,file="supervised_harmony.UMAP.rds")

42. **Integrate data using Harmony.** SingCellaR also implements a wrapper function for Harmony integration method (Korsunsky et al., 2019). Harmony can be performed using the ‘runHarmony’ function. Here, sequencing batch and donor IDs as defined are specified as covariates. UMAP analysis will be performed to obtain the embedding.
  > library(harmony)
  > SingCellaR::runHarmony(Human_HSPC,
       covariates = c("batch","donor"),
       n.dims.use = 40,
       harmony.max.iter = 20,
       n.seed = 6)
  The following parameters are required:
  a. **covariates:** The name(s) of the covariate(s) specified as batch effect to be adjusted. The names should be the same as the column names of the cell metadata.
  b. **n.dims.use:** The number of PCs as determined from step 39 to be used in this step.
  c. **harmony.max.iter:** The maximum number of rounds to run harmony. Default value is 10.
  d. **n.seed:** The random seed number generator. Default value is 1.
    > SingCellaR::runUMAP(Human_HSPC,
          useIntegrativeEmbeddings = T,
          integrative_method = "harmony",
          umap_method = "uwot",
          n.dims.use = 40,
          uwot.metric = "euclidean",
          n.seed = 1)
    The UMAP analysis result from Harmony integration will be saved. This UMAP object contains cell metadata and UMAP coordinates that will be used to compare with the results from other integrative methods.
    > harmony.UMAP<-get_umap.result(Human_HSPC)
    > saveRDS(harmony.UMAP,file="harmony.UMAP.rds")

43. **Integrate data using Seurat.** SingCellaR implements two wrapper functions for Seurat integration (Hao et al., 2021). First, the function ‘runSeuratIntegration’ is for the standard Seurat integration with Canonical Correlation Analysis (CCA). Second, the function ‘runSeuratIntegration_with_rpca’ is for the fast integration using Reciprocal PCA (RPCA). More details about Seurat integration are described on Seurat’s website (https://satijalab.org/seurat/). Due to the fast integration of using RPCA, in this protocol, we will demonstrate the function ‘runSeuratIntegration_with_rpca’ as an example. However, the user should try Seurat CCA to make a comparison of the integrative results. The user can find how to use the function ‘runSeuratIntegration’ from SingCellaR’s vignette. After the integration, the UMAP analysis from Seurat RPCA integration will be performed to obtain the embedding.
  > library(Seurat)
  > meta.data<-get_cells_annotation(Human_HSPC)
  > rownames(meta.data)<-meta.data$Cell
  > SingCellaR::runSeuratIntegration_with_rpca(Human_HSPC,
   Seurat.metadata=meta.data,
   n.dims.use = 40,
   Seurat.split.by = "data_set",
   use.SingCellaR.varGenes = T)
  The following parameters are required:
  a. **Seurat.metadata:** The cell metadata.
  b. **n.dims.use:** The number of PCs as determined from step 39 to be used in this step.
  c. **Seurat.split.by:** The indicated feature name found in the cell metadata for splitting samples for integration.
  d. **Use.SingCellaR.varGenes:** If set to TRUE, the highly variable genes identified by SingCellaR will be used. If set to FALSE, the highly variable genes will be identified using Seurat. Default value is FALSE.
    Next, the UMAP analysis from Seurat RPCA integration will be performed and saved. This UMAP object will be used to compare with the results from other integrative methods.
    > SingCellaR::runUMAP(Human_HSPC,
         useIntegrativeEmbeddings = T,
         integrative_method = "seurat",
         umap_method = "uwot",
         n.dims.use = 40,
         uwot.metric = "euclidean",
         n.seed = 1)
    > Seurat_rpca.UMAP<-get_umap.result(Human_HSPC)
    > saveRDS(Seurat_rpca.UMAP,file="Seurat_rpca.UMAP.rds")

44. **Integrate data using Scanorama.** SingCellaR implements a wrapper function for Scanorama integration (Hie et al., 2019). Scanorama can be performed using the ‘runScanorama’ function. After the integration, the standard PCA and UMAP analyses from Scanorama integration will be performed to obtain the embedding.

> runScanorama(Human_HSPC)

> runPCA(Human_HSPC,use.scanorama.integrative.matrix = T,use.components = 40)

> SingCellaR::runUMAP(Human_HSPC,

     dim_reduction_method = "pca",

     umap_method = "uwot",

     n.dims.use = 40,

     uwot.metric = "euclidean",

     n.seed = 1)

> Scanorama.UMAP<-get_umap.result(Human_HSPC)

> saveRDS(Scanorama.UMAP,file="Scanorama.UMAP.rds")

45. **Integrate data using Limma batch correction method.** To perform Limma analysis (Ritchie et al., 2015), SingCellaR provides the ‘remove_unwanted_confounders’ wrapper function to regress out the unwanted source of variation. We illustrate below for regressing out the effect from the library size, donor, and batch.

> remove_unwanted_confounders(Human_HSPC,

 residualModelFormulaStr="∼UMI_count+donor+batch")

> runPCA(Human_HSPC,use.regressout.data = T)

> SingCellaR::runUMAP(Human_HSPC,

     dim_reduction_method = "pca”,

     umap_method = "uwot",

     n.dims.use = 40,

     uwot.metric = "euclidean",

     n.seed = 1)

> Limma.UMAP<-get_umap.result(Human_HSPC)

> saveRDS(Limma.UMAP,file="Limma.UMAP.rds")

**Figure 3:**
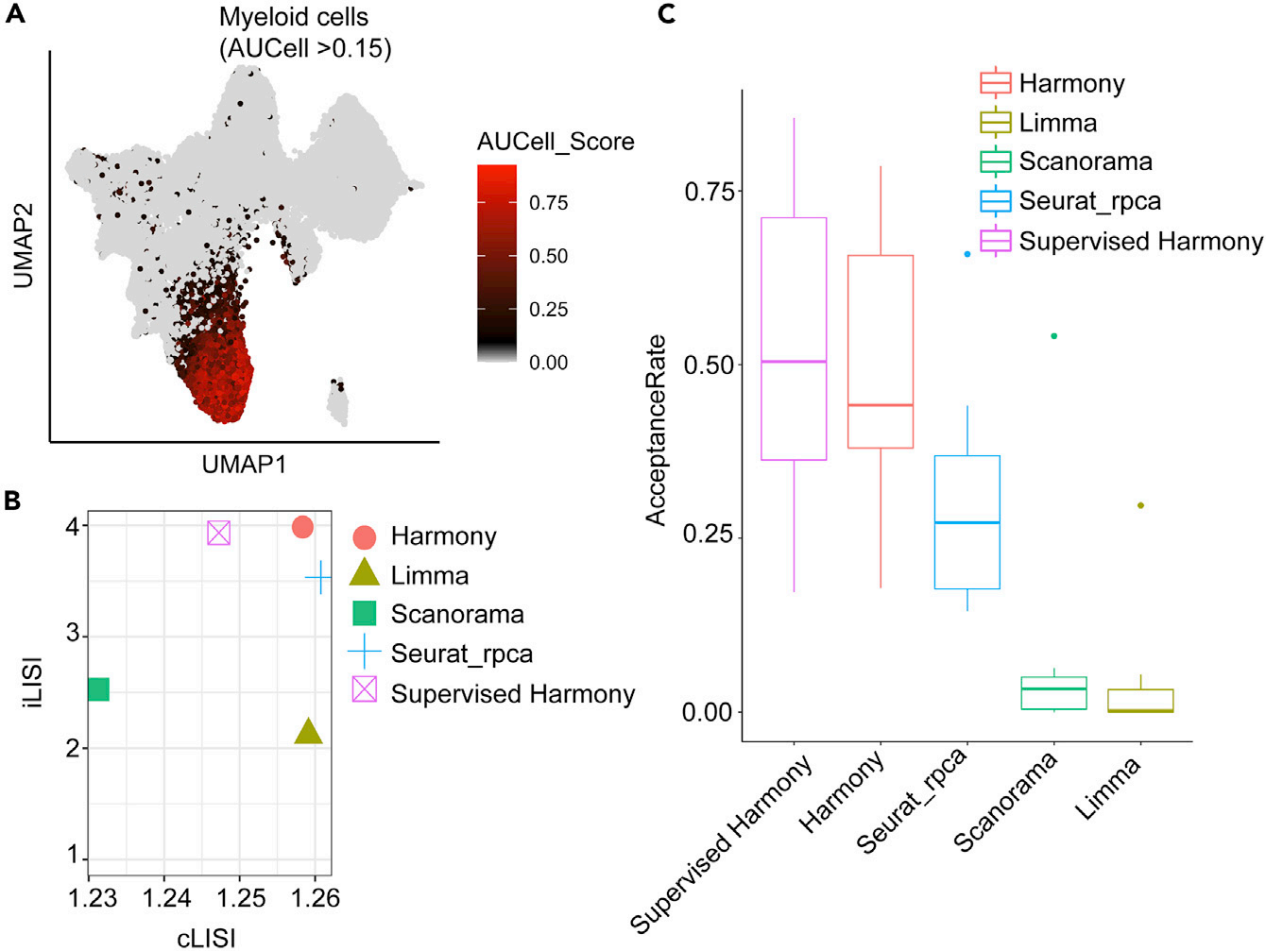
Benchmarking the integration methods (A) The UMAP plot shows AUCell scores of the cells calculated using myeloid signature genes. (B) LISI analysis on different integrative methods. X-axis represents the cLISI score and y-axis represents the iLISI score. The more accurate integration should result in a higher iLISI score and cLISI score close to 1. (C) Boxplot of kBET average acceptance rate score for each integrative method.

46. **Assign a cell type to single cells using the AUCell analysis.** In this step, we will perform AUCell analysis (Aibar et al., 2017) with seven lineage signature genes including HSC/MPP, myeloid, lymphoid, erythroid, megakaryocytic, eosinophil/basophil/mast, and endothelial progenitor cells. This step will identify the cell types that can be used for benchmarking distinct integrative methods. We assume that cells with high AUCell scores, high expression of signature genes, indicate strong ground truth of the assigned cell type. Therefore, we would expect that the same cell type should be aggregated well together when applying integrative methods.
  > library(AUCell)
  > exprMatrix <- get_umi_count(Human_HSPC)
  > human_HSPCs_cells_rankings <- AUCell_buildRankings(exprMatrix,
           nCores=4,
           plotStats=TRUE)
  The following parameters are required:
  a. **exprMat:** The raw expression count matrix. This can be retrieved from the SingCellaR object using the ‘get_umi_count’ function.
  b. **nCores:** The number of cores to use for parallel processing. The maximum number of cores is dependent on the user’s device. Default value is 1.
  c. **plotStats**: If set to TRUE (default), the expression statistics will be summarized and plotted in the histogram and boxplots.
    ***Note:*** This step may be time-consuming. The user is advised to save the output of this step using the following code:
    > save(human_HSPCs_cells_rankings,
     file="./Human_HSPC_rankings.AUCells.rdata")
    Next, the AUCell analysis will be performed using the ‘Run_AUCell’ function.
    > human_HSPCs.AUCells.score <- Run_AUCell(Human_HSPC,
     AUCell_buildRankings.file = "Human_HSPC_rankings.AUCells.rdata",
     geneSets.gmt.file = "./Human_genesets/human.signature.genes.v1.gmt")
    The following parameters are required:
  d. **AUCell_buildRankings.file:** The input file name from the AUCell rankings.
  e. **geneSets.gmt.file:** The GMT file name that contains gene sets.
    To explore the AUCell scores on UMAP plots, the user can run UMAP analysis using different types of integrative methods. This step is to identify the AUCell cut-off score for a particular cell type. The example below shows the ‘plot_umap_label_by_AUCell_score’ function that will be used to plot the myeloid AUCell scores (Figure 3A).
    > SingCellaR::runUMAP(Human_HSPC,
         useIntegrativeEmbeddings = T,
         integrative_method = "supervised_harmony",
         umap_method = "uwot",
         n.dims.use = 40,
         uwot.metric = "euclidean",
         n.seed = 1)
    >plot_umap_label_by_AUCell_score(Human_HSPC,
    AUCell_gene_set_name=c("Myeloid"),Human_HSPC.AUCells.Score,AUCell_cutoff=0.1
    5,point.size = 0.5)
    Next, cells with high AUCell scores (e.g., > 0.2 or > 0.15) for each cell type will be assigned.
    > Human_HSPC.CellType<-Human_HSPC.AUCells.Score
    > Human_HSPC.CellType$CellType<-""
    > Human_HSPC.CellType$CellType[Human_HSPC.CellType$HSPC_MPP >0.2]<-"HSC_MPP"
    > Human_HSPC.CellType$CellType[Human_HSPC.CellType$Erythroid >0.15]<-"Erythroid"
    > Human_HSPC.CellType$CellType[Human_HSPC.CellType$Myeloid >0.15]<-"Myeloid"
    > Human_HSPC.CellType$CellType[Human_HSPC.CellType$Lymphoid >0.15]<-"Lymphoid"
    > Human_HSPC.CellType$CellType[Human_HSPC.CellType$Megakaryocyte >0.15]<-
    "Megakaryocyte"
    > Human_HSPC.CellType$CellType[Human_HSPC.CellType$Eosinophil_Basophil_Mast >
    0.15]<-"Eo_Ba_Mast"
    > Human_HSPC.CellType$CellType[Human_HSPC.CellType$Endothelial_cells > 0.15]<-
    "Endothelial_cell"
    The user can explore the number of cells with high AUCell scores for each cell type using the function below and the Human_HSPC.CellType data frame object will be saved for use as the reference to perform benchmarking explained in the next step.
    > table(Human_HSPC.CellType$CellType)
    > saveRDS(Human_HSPC.CellType,file="Human_HSPC.CellType_from_AUC_High.rds")

47. **Benchmark distinct integrative methods using LISI and kBET methods.** Next, we assess whether single cells with identified cell types derived from the AUCell analysis are clustered well across covariate variables (e.g., batch and donor). SingCellaR provides the wrapper functions for a Local Inverse Simpson’s Index (LISI) (Korsunsky et al., 2019) and *k*-nearest-neighbor batch-effect test (kBET) (Buttner et al., 2019) to measure LISI and kBET scores across different integrative methods. First, the ‘runLISI’ function is performed as shown below. This function will plot LISI scores across different integrative methods (Figure 3B).
  > library(lisi)
  > reference.celltypes<-"Human_HSPC.CellType_from_AUC_High.rds"
  > integrative.umaps<-c("supervised_harmony.UMAP.rds",
     "harmony.UMAP.rds",
     "Seurat_rpca.UMAP.rds",
     "Scanorama.UMAP.rds",
     "Limma.UMAP.rds")
  > method.names<-c("Supervised Harmony","Harmony","Seurat_rpca","Scanorama",
     "Limma")
  > runLISI(lisi_label1="donor",lisi_label2="CellType",
    reference.celltypes.rds.file=reference.celltypes,
    integrative.umap.rds.files=integrative.umaps,
    integrative.method.names=method.names,IsShowPlot = T)
  The following parameters are required:
  a. **lisi_label1:** The covariate variable name of interest such as batch or donor. Default value is donor.
  b. **lisi_label2:** The variable name that represents ground truth or high AUC score cell type. Default value is CellType.
  c. **reference.celltype.rds.file:** The RDS file name that contains cell type information.
  d. **integrative.umap.rds.files:** The RDS file names that contain UMAP coordinate information generated by different integrative methods.
  e. **integrative.method.names:** The integrative method names that represent in the same order as in integrative.umap.rds.files.
  f. **IsShowPlot:** If set to TRUE (default), the iLISI and cLISI scores will be plotted.
    Second, the ‘runKBET’ function is performed as shown below. This function will calculate kBET scores across different integrative methods and return a data frame that can be used for plotting.
    > library(kBET)
    > reference.celltypes<-"Human_HSPC.CellType_from_AUC_High.rds"
    > integrative.umaps<-c("supervised_harmony.UMAP.rds",
       "harmony.UMAP.rds",
       "Seurat_rpca.UMAP.rds",
       "Scanorama.UMAP.rds",
       "Limma.UMAP.rds")
    > method.names<-c("Supervised Harmony",
        "Harmony",
        "Seurat_rpca",
        "Scanorama",
        "Limma")
    >kBET_result<-runKBET(covariate_variable_name="donor",
        reference.celltypes.rds.file=reference.celltypes,
        integrative.umap.rds.files=integrative.umaps,
        integrative.method.names=method.names,
        n.sample=1000)
    The following parameters are required:
  g. **Covariate_variable_name:** The covariate variable name of interest such as batch or donor. Default value is donor.
  h. **reference.celltype.rds.file:** The RDS file name that contains cell type information.
  i. **integrative.umap.rds.files:** The RDS file names that contain UMAP coordinate information generated by different integrative methods.
  j. **integrative.method.names:** The integrative method names that represent in the same order as in integrative.umap.rds.files.
  k. **n.sample:** The downsample size of data points used in kBET analysis. Default value is 1,000.
    ***Note:*** This step may be time-consuming, depending on the number of cells downsampled for kBET analysis.
    Next, kBET scores across integrative methods will be plotted using the ‘ggplot’ function (Figure 3C).
    > level_order <- factor(kBET_result$Method, level = c('Supervised Harmony',
    'Harmony', 'Seurat_rpca','Scanorama','Limma'))
    > ggplot(kBET_result, aes(x=level_order, y=AcceptanceRate, color=Method)) +
      + geom_boxplot()+theme_classic()+theme(axis.title.x=element_blank())
    In this step, we illustrate how to benchmark integrative results generated from different methods using the wrapper functions for LISI and kBET analyses implemented in SingCellaR. We show the objective measurement of integration for each method using iLISI and cLISI scores (Figure 3B) and kBET average acceptance rate score (Figure 3C). The user can observe from the plots that Supervised Harmony shows higher kBET and iLISI scores, and cLISI score is close to 1, indicating better data integration for this HSPC dataset compared to other methods described in (Roy et al., 2021). Therefore, the integration result from Supervised Harmony will be used for further downstream analyses.

48. **Visualize selected features and cell lineages on low dimensional space.** Here, we will assess whether the data integration was performed successfully. To this end, we annotate the UMAP by sample ID, donor type, sequencing batch, and lineage signature genes. After running the following codes, we observe that cells are clustered by cell lineage, while sample ID, donor type and sequencing batch effects are successfully corrected. These indicate that data integration and batch correction was effective in eliminating batch effect, while enabling functionally related cell to be clustered in close proximity.
  a. Annotate UMAP plot by sample ID (Figure 4A).
    > plot_umap_label_by_a_feature_of_interest(Human_HSPC,
          feature = "sampleID",
          point.size = 0.5,
          mark.feature = F)
  b. Annotate UMAP plot by donor (Figure 4B).
    > plot_umap_label_by_a_feature_of_interest(Human_HSPC,
         feature = "donor",
         point.size = 0.5,
         mark.feature = F)
  c. Annotate UMAP plot by sequencing batch (Figure 4C).
    > plot_umap_label_by_a_feature_of_interest(Human_HSPC,
          feature = "batch",
          point.size = 0.5,
          mark.feature = F)
    The following parameters are required:
    i. **feature:** The feature to annotate on UMAP plot. The feature name should match the column name of the cell metadata.
    ii. **point.size:** Size of the data points on UMAP. Default value is 1.
    iii. **mark.feature**: If set to TRUE (default), the feature name will be shown on the plot.
  d. Annotate UMAP plot by lineages genes (Figure 4D).
    > plot_umap_label_by_multiple_gene_sets(Human_HSPC,
     gmt.file = "./Human_genesets/human.signature.genes.v1.gmt",
     show_gene_sets = c("Erythroid",
     "Myeloid","Lymphoid","Megakaryocyte"),
     custom_color = c("red","cyan","orange","purple"),
     isNormalizedByHouseKeeping = F,
     point.size = 1)
    **Figure 4:**
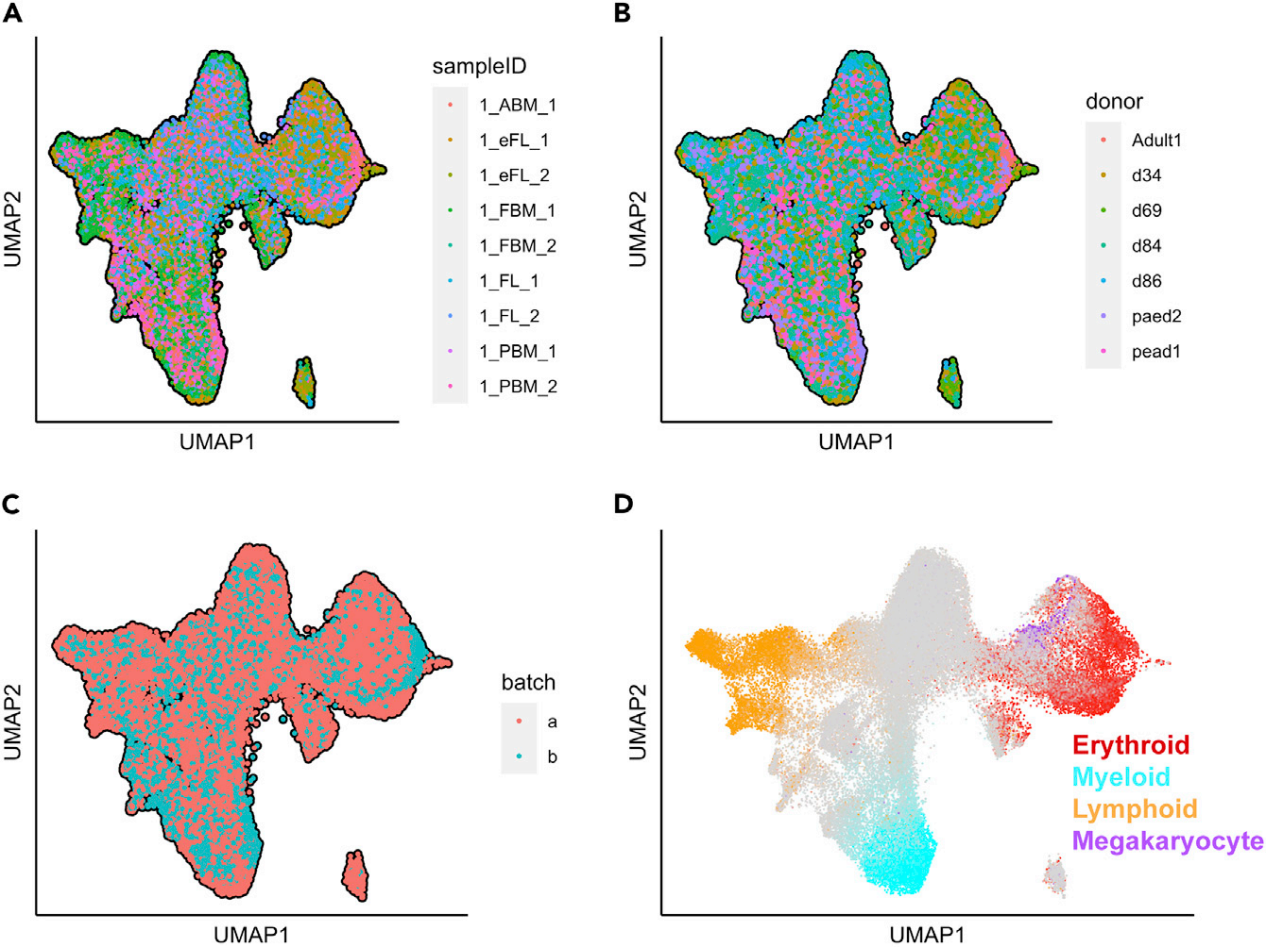
Visualization of UMAP generated from the integration of whole datasets (A–D) UMAP plots showing superimposition of (A) sample IDs; (B) donors; (C) sequencing batches; and (D) the expression of lineage gene sets. Yellow – lymphoid cells; Cyan – myeloid cells; Red – erythroid cells; Purple – megakaryocytic cells; Gray – HSPCs that do not (or lowly) express lineage signature genes.

49. Detect and assign clusters.

> identifyClusters(Human_HSPC,

    useIntegrativeEmbeddings = T,

    integrative_method = "supervised_harmony",

    n.dims.use = 40,

    knn.metric = "euclidean",

    n.neighbors = 30)

***Note:*** Information in this step and the required parameters have been detailed in step 25 with the additional ‘integrative_method’ parameter specified to indicate the data integration and batch correction method used in step 40.

**Figure 5:**
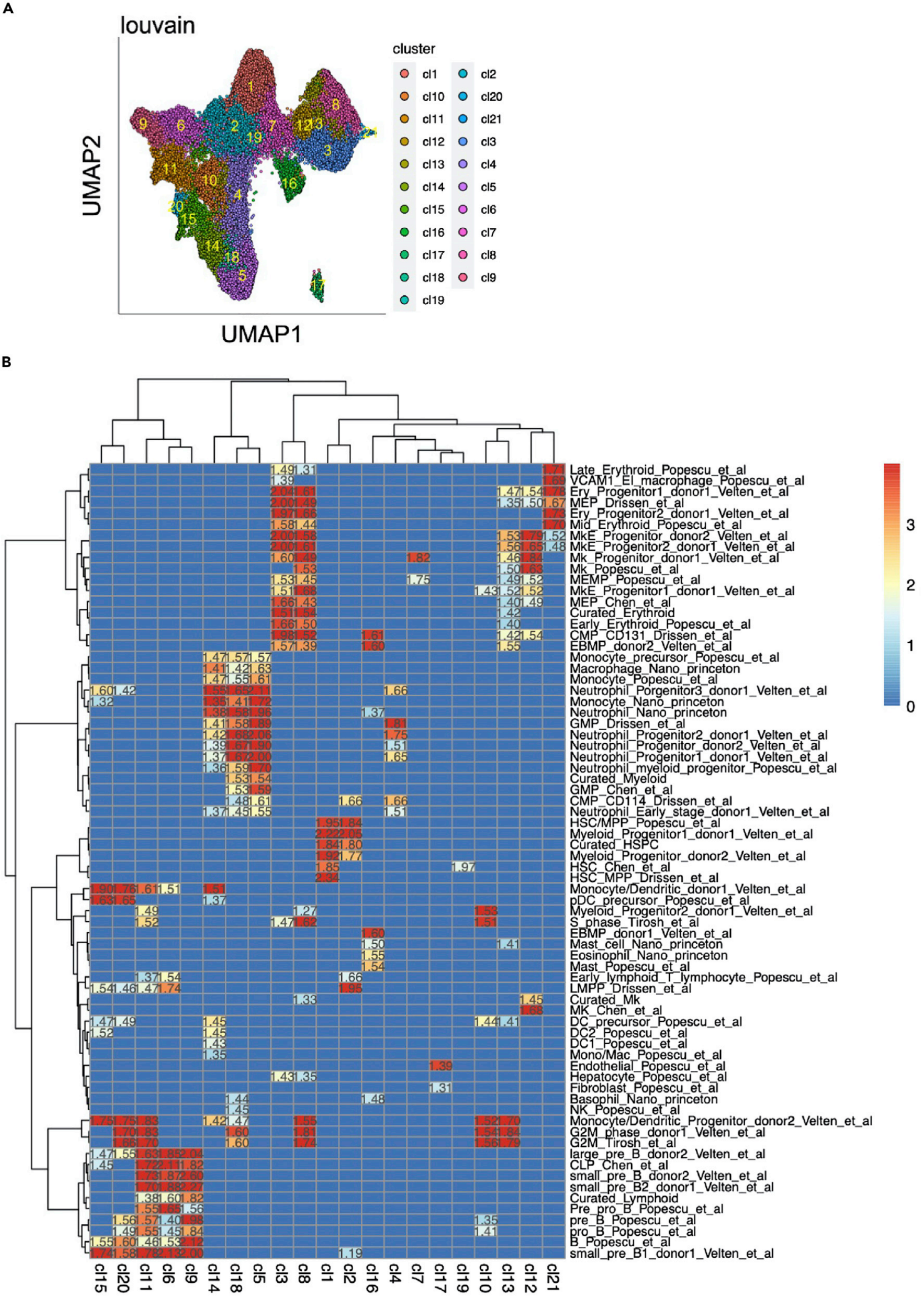
Cell clustering and annotation (A) The UMAP plot of 57,489 cells labeled with distinct 21 clusters, identified using the Louvain community-detection clustering method. (B) The heatmap showing positive gene set enrichment scores from GSEA analysis comparison of each cluster against the rest of clusters. The heatmap is generated using the function ‘plot_heatmap_for_fGSEA_all_clusters’. The x-axis represents the identified clusters depicted in (A). The y-axis represents the list of curated hematopoietic gene signatures.

50. Visualize clusters on low dimensional space. (Figure 5A).

> plot_umap_label_by_clusters(Human_HSPC,

   show_method = "louvain",

   mark.clusters = T)

51. **Identify cluster-specific genes.** This step will perform differential gene expression analysis to identify marker genes per each cluster.

> findMarkerGenes(Human_HSPC,cluster.type = "louvain")

***Note:*** See details in step 27. This step will take time to run on the fully integrated datasets depending on the number of cells and identified clusters.

52. **Save the integrated R object for further analyses.**

> save(Human_HSPC,

  file="./Human_HSPC_All.SingCellaR.rdata")

**Pause point:** The user can save the integrative SingCellaR_Int object for further downstream analyses.

### Cell type annotation

**Timing: 1.5–2 h**

The aim of cell type annotation is to assign a cell type identity to each cluster. The expression of selected marker genes can be visualized and explored across the different clusters, either using UMAP, dotplot, heatmap, or violin plots (Satija et al., 2015). Nevertheless, it is only feasible to explore a small number of gene markers using these approaches. Moreover, closely related cell populations with overlapping or subtle differences in transcriptomic profiles can be better resolved using gene sets, rather than individual genes. To more systematically and objectively annotate the clusters, SingCellaR implements a GSEA-based cell type annotation approach whereby cluster-specific ranked genes are subjected to GSEA analysis using a comprehensive list of curated gene sets. Using this approach, for each cluster, the list of genes previously included for differential expression analysis using findMarkerGenes is ranked from the most up-regulated to most down-regulated. Next, the individual genes in each curated gene set are assessed for enrichment in this ranked list of genes. Gene sets that are found higher up in the ranked list of genes are considered to be more enriched in a given cluster (Subramanian et al., 2005). This assessment of gene set enrichment is implemented by the fgsea (Korotkevich et al., 2021) package. These curated gene sets encompass 75 hematopoietic cell types and are available at Zenodo: https://doi.org/10.5281/zenodo.5879071 in GMT file format and are also available in Table S3 from the original publication (Roy et al., 2021).

53. Load the SingCellaR package.

> library(SingCellaR)

54. Load the integrated R object generated from step 52.

> load(file = "./Human_HSPC_All.SingCellaR.rdata")

**Figure 6:**
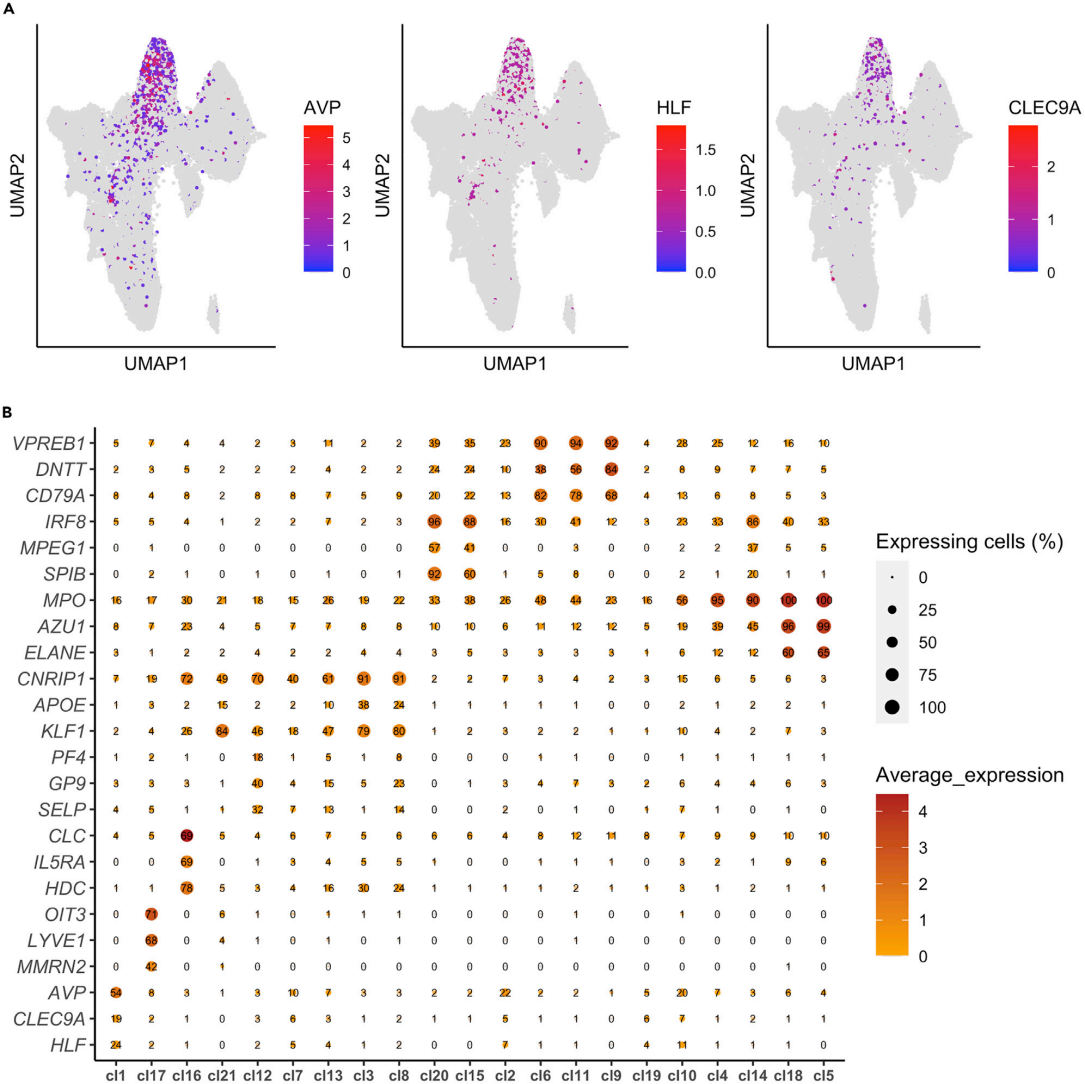
Gene expression of canonical lineage signatures and marker gene identification for each cluster (A) UMAP plots of known lineage marker genes for HSC/MPP. Red – highly expressed and blue – lowly expressed. (B) Bubble plots of known lineage marker gene expression for each cluster. The size of the dots represents the percentage of expressing cells as indicated within the dot. The x-axis represents the identified clusters.

55. **Generate the pre-ranked genes.** For each cluster, differential gene expression analysis is performed (Giustacchini et al., 2017) against all other clusters and the resulting genes are ranked based on their log2 fold change multiplied by -log10 of the adjusted *P*-value. This is to yield a more robust ranking compared to using the log2 fold change alone for the genes for GSEA. Here, the ‘identifyGSEAPrerankedGenes_for_all_clusters’ function is performed to provide a suitable format for GSEA analysis and includes all possible expressed genes above the lower cut-off parameters defined using the min.expFraction and min.log2FC arguments.
  > pre_rankedGenes_for_GSEA <-
   identifyGSEAPrerankedGenes_for_all_clusters(Human_HSPC,
        cluster.type = "louvain")
  The following parameter is required:
  a. **cluster.type**: The clustering method name.
  b. **fishers_exact_test**: The cut-off *p*-value. Default value is 0.1.
  c. **min.expFraction**: The fraction of expressing cells, above which, the gene will be included for GSEA. Default value is 0.01.
  d. **min.log2FC**: The log2 fold change, above which, the gene will be included for GSEA. Default value is 0.1.
    ***Note:*** The processing time of this step depends on the number of cells and clusters. The user is advised to save the output of this step using the following code:
    > save(pre_rankedGenes_for_GSEA,
     file="./Human_HSPCs_preRankedGenes_for_GSEA.rdata")

56. **Perform GSEA.** For each cluster, the ranked genes are subjected to GSEA to assess the enrichment for all curated hematopoietic gene sets.
  > fgsea_Results <- Run_fGSEA_for_multiple_comparisons(
   GSEAPrerankedGenes_list = pre_rankedGenes_for_GSEA,
   gmt.file = "./Human_genesets/human.hematopoiesis.signature.genes.gmt")
  The following parameters are required:
  a. **GSEAPrerankedGenes_list:** The object containing the ranked genes for each cluster generated from step 55.
  b. **gmt.file:** Curated gene sets in GMT file format.
    ***Note:*** Here, we curated gene sets encompassing 75 hematopoietic signatures, but the user can also generate other customized gene sets in GMT file format as the input for GSEA. Each line of the GMT file represents one gene set. Specifically, the first column represents the name of the gene set, the second column represents the description of the gene set, and the third column onwards represents the genes that constitute the gene set, whereby each column represents one gene. The GMT file should be saved in tab-delimited format.

57. **Visualize GSEA results.** A heatmap is used to observe and compare enrichment scores of each gene set (rows) across all clusters (columns). This visualization allows the user to annotate a cell type identity and cell states to each cluster based on the degree of enrichment of the curated gene sets. (Figure 5B).
  > plot_heatmap_for_fGSEA_all_clusters(fgsea_Results,
      isApplyCutoff = TRUE,
      use_pvalues_for_clustering=T,
      show_NES_score = T,
      fontsize_row = 5,
      adjusted_pval = 0.10,
      show_only_NES_positive_score = T,
      format.digits = 3,
      clustering_method = "ward.D",
      clustering_distance_rows = "euclidean",
      clustering_distance_cols = "euclidean",
      show_text_for_ns = F)
  The following parameters are required:
  a. **isApplyCutoff:** If set to TRUE, only the normalized enrichment scores (NES) of gene sets with adjusted *P*-values below the user-defined values in ‘adjusted_pval’ argument will be displayed on the heatmap. Default is FALSE.
  b. **use_pvalues_for_clustering:** If set to TRUE (default), the -log10(*P*-values) will be used instead of NES to cluster rows and/or columns.
  c. **show_NES_score:** If set to TRUE (default), NES will be displayed on the heatmap.
  d. **fontsize_row:** The font size of the gene set names along the rows of the heatmap. Default value is 5.
  e. **adjusted_pval:** The value, below which, NES will be displayed on the heatmap. The default value is 0.25.
  f. **show_only_NES_positive_score**: If set to TRUE, only NES > 0 will be displayed on the heatmap. Default is FALSE.
  g. **format.digits:** The number of significant digits to be used for numeric display on the heatmap. Default value is 2.
  h. **clustering_method**: The clustering method for clustering the rows and/or columns. Default is "complete".
  i. **clustering_distance_rows:** The distance metric to use when clustering the rows. Default is "euclidean".
  j. **clustering_distance_cols:** The distance metric to use when clustering the columns. Default is "euclidean".
  k. **show_text_for_ns**: If set to TRUE (default), non-significant (ns) NES will be displayed on the heatmap.

58. **Visualize selected canonical marker genes using UMAP.** One or more individual genes can be plotted on UMAP using the ‘plot_umap_label_by_genes’ function (Figure 6A).
  # HSC/MPP
  > plot_umap_label_by_genes(Human_HSPC,gene_list = c("AVP","HLF","CLEC9A"))
  # Myeloid progenitor
  > plot_umap_label_by_genes(Human_HSPC,gene_list = c("ELANE","MPO","AZU1"))
  # Erythroid progenitor
  > plot_umap_label_by_genes(Human_HSPC,gene_list = c("KLF1","CNRIP1","APOE"))
  # Megakaryocytic progenitor
  > plot_umap_label_by_genes(Human_HSPC,gene_list = c("PF4","GP9","SELP"))
  # B lymphoid progenitor
  > plot_umap_label_by_genes(Human_HSPC,gene_list = c("DNTT","CD79A","VPREB1"))
  # Dendritic precursor
  > plot_umap_label_by_genes(Human_HSPC,gene_list = c("SPIB","IRF8","MPEG1"))
  # Eosinophil/Basophil/Mast progenitor
  > plot_umap_label_by_genes(Human_HSPC,gene_list = c("CLC","HDC","IL5RA"))
  # Endothelial cells
  > plot_umap_label_by_genes(Human_HSPC,gene_list = c("OIT3","MMRN2","LYVE1"))
  The following parameter is required:
  a. **gene_list:** A vector of one or more gene names to plot.

59. **Visualize selected canonical marker genes using bubble plot** (Figure 6B). One or more individual genes can be plotted using the ‘plot_bubble_for_genes_per_cluster’ function. Each gene will be represented on each row of the output.
  > marker.genes <- c("AVP","HLF","CLEC9A",
     "PF4","GP9","SELP",
     "KLF1","CNRIP1","APOE",
     "ELANE","MPO","AZU1",
     "DNTT","CD79A","VPREB1",
     "SPIB","IRF8","MPEG1",
     "CLC","HDC","IL5RA",
     "OIT3","MMRN2","LYVE1")
  > plot_bubble_for_genes_per_cluster(Human_HSPC,
       cluster.type = "louvain",
       gene_list = marker.genes,
       show.percent = TRUE)
  The following parameters are required:
  a. **cluster.type:** The clustering method name used to identify and assign the cell clusters.
  b. **gene_list:** A vector of one or more gene names to plot.
  c. **show.percent:** If set to TRUE, the percentage of expressing cells for respective genes in each cluster are displayed on the dotplot. Default is FALSE.

60. **Visualize identified marker genes for each cluster using heatmap.** One or more individual genes can be plotted using a heatmap with the ‘plot_heatmap_for_marker_genes’ function. Each gene is represented on each row of the output.
  > plot_heatmap_for_marker_genes(Human_HSPC,
      cluster.type = "louvain",
      n.TopGenes = 8)
  The following parameters are required:
  a. **cluster.type:** The name of the clustering method used to identify and assign the cell clusters.
  b. **n.TopGenes:** The number of top genes for each cluster to plot. Default value is 5.

61. **Export top marker genes for each cluster.** The top marker genes with statistical analysis results can be exported to the text file format.
  > export_marker_genes_to_table(Human_HSPC,
      cluster.type = "louvain",
      n.TopGenes = 100,
      min.log2FC = 0.5,
      min.expFraction = 0.3,
      write.to.file =
    "./Human_HSPC_marke_genes_per_cluster.txt")
  The following parameters are required:
  a. **cluster.type:** The clustering method name used to identify and assign the cell clusters.
  b. **n.TopGenes:** The number of top genes for each cluster. Default value is 5.
  c. **min.log2FC:** The log2FC value, above which, genes will be included. Default value is 0.5.
  d. **min.expFraction:** The fraction of expressing cells, above which, genes will be included. Default value is 0.3.
  e. **write.to.file:** The file path to be exported.

### AUCell analysis

**Timing: 2–3 h**

The user can calculate the enrichment of specific gene sets (e.g., lineage signature and cell cycle genes) assigned for an individual cell. We incorporated AUCell score analysis (Aibar et al., 2017) into SingCellaR to assign and define high-confident lineage-specific cells. This cell-level enrichment analysis enables us to validate our cell type annotation and check for the presence of heterogeneous cell populations within a given cluster. This can be further used to compare differential abundance of different cell lineages across developmental stages.

62. **Load SingCellaR and required R packages.** Here, the user can load the integrated R object saved from step 52.

> library(SingCellaR)

> library(AUCell)

> library(ggplot2)

> library(DAseq)

> source('./utilis.R')

63. Load the integrated R object generated from step 52.

> load(file = "./Human_HSPC_All.SingCellaR.rdata")

64. **Build AUCell gene rankings.** The user will have to create the ranked gene list using the function ‘AUCell_buildRankings’ implemented in AUCell package.
  > set.seed(2021)
  > exprMatrix <- get_umi_count(Human_HSPC)
  > human_HSPCs_cells_rankings <- AUCell_buildRankings(exprMatrix,
          nCores=4,
         plotStats=TRUE)
  The following parameters are required:
  a. **exprMat:** The raw expression count matrix. This can be retrieved from the SingCellaR object using the ‘get_umi_count’ function.
  b. **nCores:** The number of cores to use for parallel processing. The maximum number of cores is dependent on the user’s device. Default value is 1.
  c. **plotStats:** If set to TRUE (default), the expression statistics will be summarized and plotted in the histogram and boxplots.
    ***Note:*** This step may be time-consuming. The user is advised to save the output of this step using the following code:
    > save(human_HSPCs_cells_rankings,
     + file="./Human_HSPC_rankings.AUCells.rdata")

65. **Calculate AUCell scores.** AUCell scores for each cell will be computed using the ranked genes from the previous step for the provided hematopoietic gene sets.

> set.seed(2021)

> human_HSPCs.AUCells.score <- Run_AUCell(Human_HSPC,

  AUCell_buildRankings.file = "Human_HSPC_rankings.AUCells.rdata",

  geneSets.gmt.file = "./Human_genesets/human.signature.genes.v1.gmt")

***Optional:*** The user is advised to save the AUCell scores for further analysis.

> save(human_HSPCs.AUCells.score,

 file="./human_HSPCs.AUCells.score.rdata")

**Figure 7:**
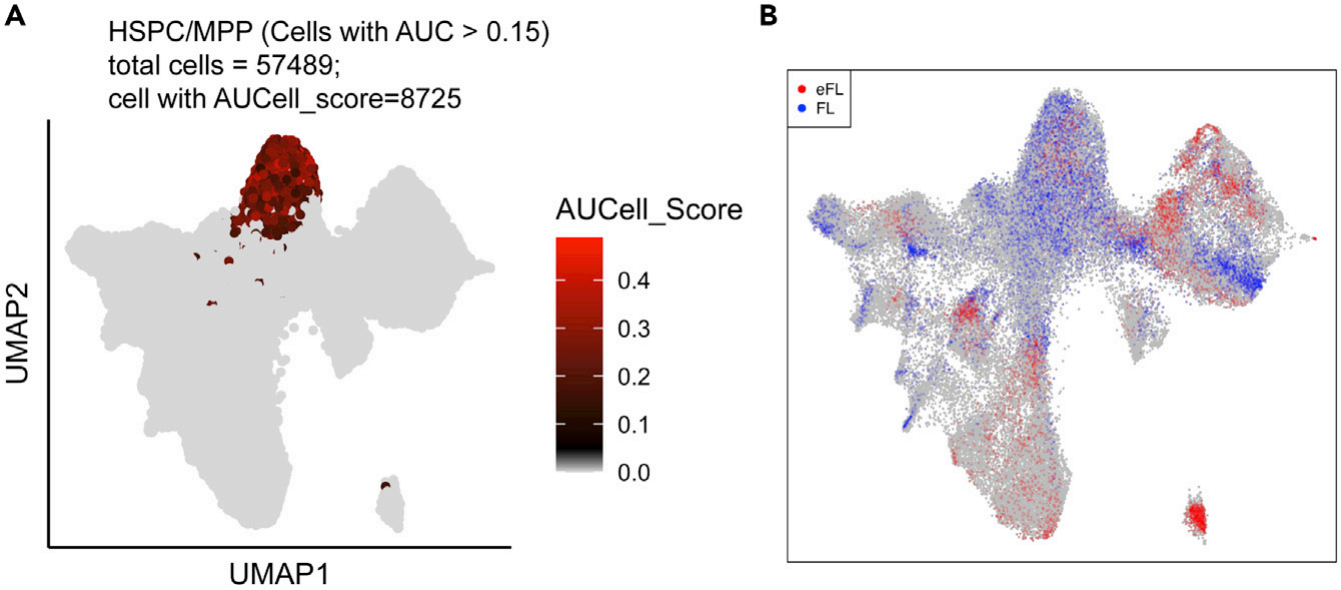
UMAP plots overlaid with computed scores from AUCell and differential abundance analyses (A) The UMAP plot shows AUCell scores of the cells calculated using the HSC/MPP signature genes. Cells with the AUCcell score greater than 0.15 within cluster 1 are highlighted with gradient colors from low (black) to high (red) scores. (B) The UMAP plot shows the differential abundance scores from the logistic classifier prediction calculated from DAseq analysis of pairwise comparison between eFL *vs.* FL. Cells in red are predicted to be more abundant in eFL, whereas cells in blue indicate higher abundance in FL. Cells in gray do not have substantially different abundance score.

66. **Visualize AUCell scores.** The user can visualize AUCell scores for a given gene signature on specific clusters on the UMAP embedding. Here, we will use HSC/MPP gene signature on cluster 1 as an example (Figure 7A). We observe HSC/MPP gene signature to be uniformly enriched across majority of cells from this cluster. This is consistent with our cluster-level GSEA results that indicate cluster 1 is enriched for several HSC and MPP gene sets.
  > plot_umap_label_by_AUCell_score(Human_HSPC,
       AUCell_gene_set_name = "HSPC_MPP",
       AUCell_score = human_HSPCs.AUCells.score,
       selected.limited.clusters = "cl1",
       IsLimitedAUCscoreByClusters = T,
       AUCell_cutoff = 0.15)
  The following parameters are required:
  a. **AUCell_gene_set_name:** The name of the gene signature to plot. The signature name specified here must be the same as the gene signature name in the GMT file provided in step 65.
  b. **AUCell_score:** The R object created from computing AUCell scores in step 65.
  c. **selected.limited.clusters:** Cells in selected cluster IDs will be displayed with the AUCell scores.
  d. **IsLimitedAUCscoreByClusters:** If set to TRUE, the AUCell scores will only be displayed for selected clusters as specified using the ‘selected.limited.clusters’ argument. Default is FALSE.
  e. **AUCell_cutoff:** The AUCell score threshold, above which, the scores will be displayed. The higher the score threshold, the more stringent the threshold. Default is 0.
    ***Note:*** AUCell cutoff score is arbitrary. To explore the AUCell cutoff score for each gene signature, the user can plot the score distribution using ggplot2 and manually explore the suitable threshold.

67. **Differential abundance testing.** We use DAseq (Zhao et al., 2021) to perform pairwise comparisons for cellular abundance in different tissues across developmental stages. We have incorporated the DAseq analysis into the ‘run_DAseq_comparison’ function. Here, we will show the differential abundance of cell populations between eFL and FL (Figure 7B) as an example. Erythroid progenitors are enriched in FL, whereas lymphoid and myeloid progenitors are predominantly derived from BM samples. Marked differences are also observed for megakaryocytic progenitors whereby these cells are mainly from eFL.
  > run_DAseq_comparison(Human_HSPC,
      groupA = "eFL",
      groupB = "FL",
      labels.1 = c("eFL_1","eFL_2"),
      labels.2 = c("FL_1","FL_2"),
      path = "./",
      outputname = "eFL_vs_FL.pdf")
  The following parameters are required:
  a. **groupA** and **groupB:** The group names for integrated samples (e.g., eFL, FL, FBM, and PBM) or an individual sample name.
  b. **labels.1** and **labels.2:** The sample IDs of groupA and groupB, respectively.
  c. **path:** The folder path to save the output file.
  d. **outputname:** The output file name.

### Trajectory analysis

**Timing: 1–1.5 h**

From the annotation of cell types assigned in the cell type annotation steps, the user can further investigate the direction of cellular differentiation trajectories. The user can trace immature to more differentiated cell populations and understand the functional relationship between different cell populations in terms of cellular maturity. It is noteworthy that current trajectory analysis requires the user to identify the starting point (immature cell state), in this case, HSC/MPP. Hence, the cell annotations inferred from previous steps will be helpful to infer the primitive cell clusters. SingCellaR supports two approaches of trajectory analysis, namely force-directed graph (FDG) (Jacomy et al., 2014) and diffusion map (Haghverdi et al., 2015). We will also perform Monocle3 analysis (Trapnell et al., 2014), a pseudotime-based method (Haghverdi et al., 2015) to infer cellular trajectories.

### Method 1: Force-directed graph analysis

68. **Load SingCellaR package.**

> library(SingCellaR)

69. **Load the integrated R object generated from step 52.**

> load(file = "./Human_HSPC_All.SingCellaR.rdata")

**Figure 8:**
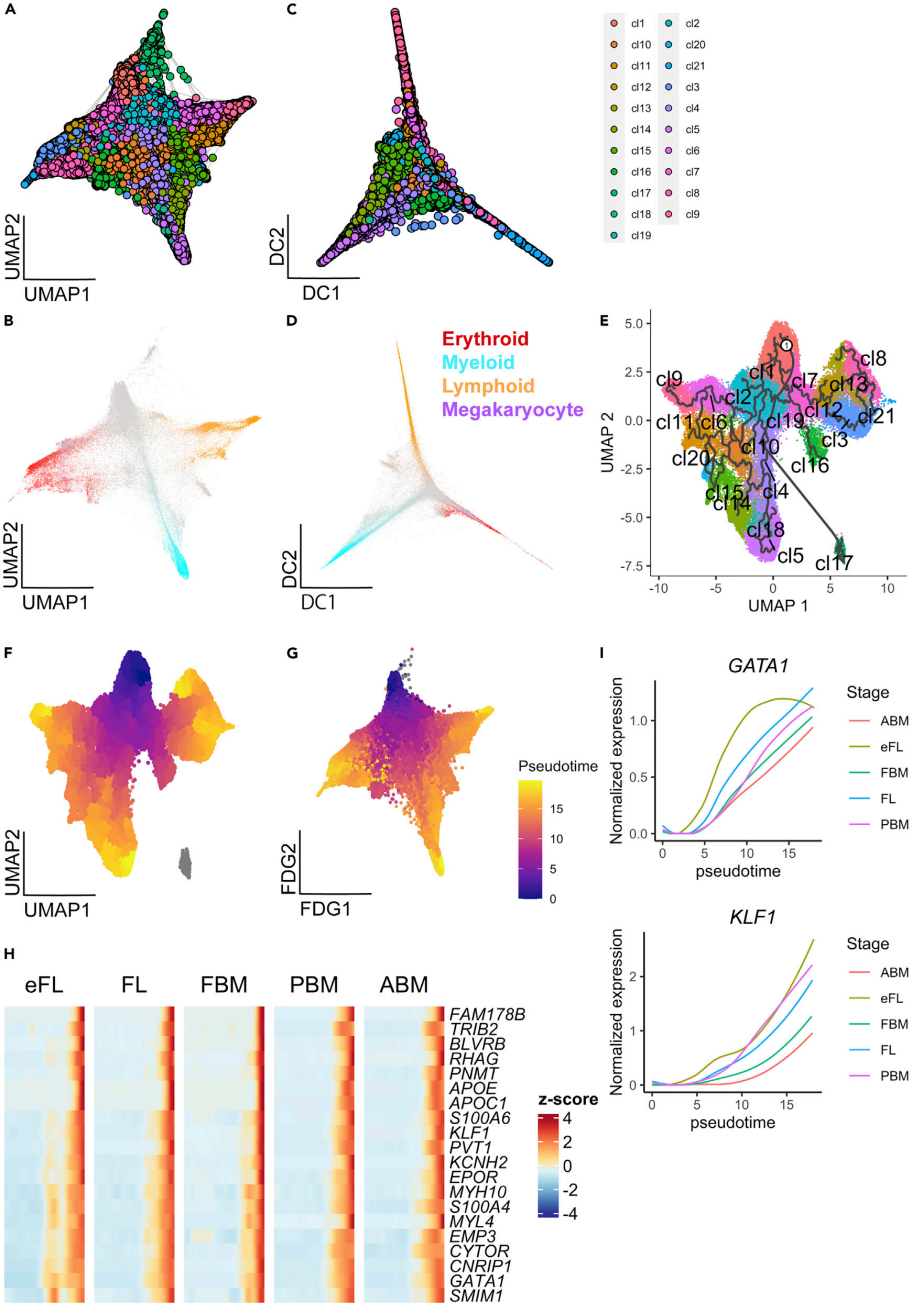
Differentiation trajectory analysis using the combination of SingCellaR and Monocle3 (A and B) The force-directed graph displays 21 of Louvain clusters and (B) with superimposition of lineage gene scores for four lineage gene sets. Yellow – lymphoid cells; Cyan – myeloid cells; Red – erythroid cells; Purple – megakaryocytic cells; Gray – HSPCs that do not (or lowly) express lineage signature genes. (C and D) Diffusion maps of 21 Louvain clusters and with lineage gene scores in (D). (E and F) UMAP plots with identified clusters and trajectory paths using Monocle3 (E) and overlaid with pseudotime analysis scores (F). (G) Force-directed graph (FDG) with superimposition of pseudotime analysis scores. (H) Gene expression of selected lineage marker genes along pseudotime from HSC to erythroid trajectory representing across tissues. (I) The gene expression of chosen erythroid lineage genes, *GATA1* and *KLF1*, along the pseudotime of HSC to erythroid trajectory.

70. **Run force-directed graph analysis.** The user can use the ‘runFA2_ForceDirectedGraph’ function to build the force-directed graph layout (embeddings). The layout can be annotated using various features, including cell lineage signature genes. Here, we use the Supervised harmony embeddings to generate force-directed graph layout.
  > runFA2_ForceDirectedGraph(Human_HSPC,
      useIntegrativeEmbeddings = T,
      integrative_method = "supervised_harmony",
      knn.metric = "euclidean",
      n.dims.use = 40,
      n.neighbors = 5,
      n.seed = 35,
      fa2_n_iter = 1000)
  The following parameters are required:
  a. **useIntegrativeEmbeddings:** If set to TRUE, the data integration or batch correction embeddings will be used in conjunction with ‘integrative_method’ argument. Default is FALSE.
  b. **integrative_method:** The data integration or batch correction method name.
  c. **knn.metric:** The distance metric.
  d. **n.dims.use:** The number of PCs from ‘integrative_method’. If ‘useIntegrativeEmbedding’ is set to FALSE, the PCA analysis result is used. Default value is 30.
  e. **n.neighbors:** The number of neighboring cells.
  f. **n.seed:** The random number generator. Default value is 1.
  g. **fa2_n_iter:** The number of iterations for analyzing the ‘networkx’ graph. Default value is 1,000.

71. Visualize trajectories by Louvain clustering (Figure 8A).
  > plot_forceDirectedGraph_label_by_clusters(Human_HSPC, show_method =
  "louvain")
  The following parameter is required:
  a. **show_method**: The clustering method name.

72. Visualize trajectories by using multiple lineages gene sets (Figure 8B).
  > plot_forceDirectedGraph_label_by_multiple_gene_sets(Human_HSPC,
    gmt.file = "./Human_genesets/human.signature.genes.v1.gmt",
   show_gene_sets = c("Erythroid","Myeloid","Lymphoid",
     "Megakaryocyte"),
    custom_color = c("red","cyan","orange","purple"),
    isNormalizedByHouseKeeping = F,
   edge.size=0,
   edge.color = "#FFFFFF",
   vertex.size = 0.2,
   showEdge = F,
   showLegend = T)
  The following parameters are required:
  a. **gmt.file:** Path to the file containing the gene signatures in GMT format.
  b. **show_gene_sets:** The vector of gene signature names to show on the plot. The names must be the same names as found in the ‘gmt.file’.
  c. **custom_color:** The assigned colors for gene signatures in ‘show_gene_set’.
  d. **isNormalizedByHouseKeeping:** When set to TRUE (default), the gene expression values of each gene signature specified will be normalized by the housekeeping genes. The housekeeping genes are defined as the top 100 genes with the highest total gene expression values across all cells.
  e. **edge.size:** The size of the edges connecting the nodes. Default value is 0.2.
  f. **edge.color:** The color of the edges. Default color is gray.
  g. **vertex.size:** The size of the nodes. Default value is 1.5.
  h. **showEdge:** When set to TRUE (default), the edges will be displayed.
  i. **showLegend:** When set to TRUE (default), the legend will be displayed.

### Method 2: Diffusion map analysis

73. Load SingCellaR and destiny R packages.

> library(SingCellaR)

> library(destiny)

74. Load the integrated R object generated from step 52.

> load(file = "./Human_HSPC_All.SingCellaR.rdata")

75. **Run diffusion map analysis.** The user can use the ‘runDiffusionMap’ function to generate the diffusion map layout (embeddings). The layout can be annotated using various features, including cell lineage signature genes. We will use the Supervised harmony embeddings to generate the diffusion map layout.
  > runDiffusionMap(Human_HSPC,
     useIntegrativeEmbeddings = T,
     integrative_method = "supervised_harmony",
     n.dims.use = 40,
     n.seed = 10)
  The following parameters are required:
  a. **useIntegrativeEmbeddings:** If set to TRUE, the data integration or batch correction embeddings will be used in conjunction with ‘integrative_method’ argument. Default is FALSE.
  b. **integrative_method:** The data integration or batch correction method name.
  c. **n.dims.use:** The number of PCs from ‘integrative_method’. If ‘useIntegrativeEmbedding’ is set to FALSE, the PCA result will be used. Default value is 30.
  d. **n.seed:** The random number generator. Default value is 1.

76. Visualize trajectories by Louvain clustering (Figure 8C).
  > plot_diffusionmap_label_by_clusters(Human_HSPC, show_method = "louvain")
  The following parameter is required:
  a. **show_method**: The clustering method name.

77. Visualize trajectories by multiple lineages genes (Figure 8D).
  > plot_diffusionmap_label_by_multiple_gene_sets(Human_HSPC,
    gmt.file = "./Human_genesets/human.signature.genes.v1.gmt",
    show_gene_sets = c("Erythroid","Myeloid","Lymphoid","Megakaryocyte",
      "Endothelial_cells"),
    custom_color = c("red","cyan","orange","purple","green"),
    isNormalizedByHouseKeeping = F)
  The following parameters are required:
  a. **gmt.file:** Path to the file containing the gene signatures in GMT format.
  b. **show_gene_sets:** The vector names of gene signatures to show in the plot. The names must be the same names as found in the gmt.file.
  c. **custom_color:** The assigned colors for gene signatures in ‘show_gene_set’.
  d. **isNormalizedByHouseKeeping:** When set to TRUE (default), the gene expression values of the individual genes of each gene signature specified will be normalized by the housekeeping genes. The housekeeping genes are defined as the top 100 genes with the highest total gene expression values across all cells.

### Method 3: Monocle3 analysis

78. Load SingCellaR and required R packages.

> library(SingCellaR)

> library(monocle3)

> library(ggplot2)

> library(ComplexHeatmap)

> library(circlize)

> library(RColorBrewer)

> source('./utilis.R')

79. Load the integrated R object generated from step 52.

> load(file = "./Human_HSPC_All.SingCellaR.rdata")

80. **Prepare input files for Monocle3.** The required objects include the expression matrix of raw counts, cell cluster metadata, and gene metadata.

# Expression matrix

> cells.used <- Human_HSPC@sc.clusters$Cell

> umi <- get_umi_count(Human_HSPC)

> used.umi <- umi[,cells.used]

> expression_matrix <- used.umi

> dim(expression_matrix) # check the dimension of object

# Cell cluster metadata

> cell_metadata <- Human_HSPC@sc.clusters

> rownames(cell_metadata) <- cell_metadata$Cell

# Gene metadata

> gene_annotation <- as.data.frame(rownames(used.umi))

> colnames(gene_annotation) <- "gene_short_name"

> rownames(gene_annotation) <- gene_annotation$gene_short_name

81. **Create Monocle3 object.**

> cds <- new_cell_data_set(expression_data = expression_matrix,

     cell_metadata = cell_metadata,

     gene_metadata = gene_annotation)

82. **Integrate Monocle3 and SingCellaR results.** Monocle3 normalizes the raw gene counts, and then performs PCA. The user can run the default workflow as suggested by Monocle3 tutorial. In this step, we will replace Monocle3’s UMAP embeddings and add cluster information derived from the SingCellaR object.

# Pre-process Monocle3 object

> cds <- preprocess_cds(cds,num_dim = 100,method = "PCA")

> cds <- align_cds(cds)

# Substitute Monocle3's embeddings with SingCellaR's embeddings

> embeddings <- >Human_HSPC@SupervisedHarmony.embeddings

> cds@int_colData@listData$reducedDims$Aligned <- embeddings

# Nonlinear dimension reduction

> cds <- reduce_dimension(cds,

     reduction_method = "UMAP",

     umap.min_dist = 0.3,

     preprocess_method = "Aligned")

# Identify and assign clusters

> cds <- cluster_cells(cds,

   reduction_method = "UMAP",

   k = 30,

   cluster_method = "louvain")

# Substitute Monocle3's UMAP embeddings with SingCellaR's embedding

> newcds<- cds # change monocle3 objects name

> SingCellaR.umap <-,c("Cell","UMAP1","UMAP2")]

> Human_HSPC@umap.result[monocle3.umap <- newcds@int_colData$reducedDims$UMAP

> umap <- SingCellaR.umap[match(rownames(monocle3.umap),SingCellaR.umap$Cell),]

> rownames(umap) <- umap$Cell

> umap$Cell <- NULL

> newcds@int_colData$reducedDims$UMAP <- umap

# Substitute Monocle3's cluster identity with SingCellaR's cluster identity

> anno.clusters <- Human_HSPC@sc.clusters$louvain_cluster

> names(anno.clusters) <- Human_HSPC@sc.clusters$Cell

> newcds@clusters$UMAP$clusters <- anno.clusters

83. **Generate trajectory graph and order cells by pseudotime.** To learn the cell differentiation trajectories, the user will use the ‘learn_graph’ function provided by Monocle3. By default, Monocle3 uses a 'self-defined' node to perform the pseudotime analysis. Thus, the user will need to define the root node, i.e., the most immature cluster. To identify the root node, the user can use the ‘get_earliest_principal_node’ function. Based on the previous analyses, the user can select ‘cl1’, the HSC/MPP cluster, as the starting point of the trajectory.

> newcds <- learn_graph(newcds)

# Apply function to retrieve root node

> root.nodes <- get_earliest_principal_node(newcds,cluster = "cl1")

# Order cells by pseudotime relative to root node

> newcds <- order_cells(newcds, root_pr_nodes = root.nodes)

# Save R object

> save(newcds,file = "./Human_HSPC_monocle3.rdata")

84. **Visualize trajectory paths on UMAP** (Figure 8E).

> plot_cells(newcds,

   group_label_size = 5,

   color_cells_by = "louvain_cluster",

   show_trajectory_graph = T,

   label_roots = T,

   label_cell_groups = T,

   label_groups_by_cluster = T,

   label_leaves = F,

   label_branch_points = F)

85. **Visualize pseudotime on UMAP** (Figure 8F).

> plot_cells(newcds,

   color_cells_by = "pseudotime",

   show_trajectory_graph = F,

   label_roots = T,

   label_cell_groups=F,

   label_leaves=F,

   label_branch_points=F,

   graph_label_size=1.5,

   group_label_size=4,

   cell_size=1.5)

86. **Visualize pseudotime on SingCellaR FDG** (Figure 8G). Before plotting FDG, the user can remove the endothelial cells (cl17).

# Remove endothelial cell cluster

> sc.clusters <- Human_HSPC@sc.clusters[!(Human_HSPC@sc.clusters$louvain_cluster == "cl17"),]

> fa2 <- Human_HSPC@fa2_graph.layout

> fa2.used <- fa2[rownames(fa2) %in% sc.clusters$Cell,]

# Extract the pseudotime information

> new_data <- data.frame(pseudotime = pseudotime(newcds,reduction_method = "UMAP"))

> new_data$Cell <- rownames(new_data)

> new_data <- new_data[new_data$Cell %in% rownames(fa2.used),]

# Integrate pseudotime with FDG embeddings

> fa2.used <- fa2.used[match(new_data$Cell,rownames(fa2.used)),]

> colnames(fa2.used) <- c("FDG1","FDG2")

> fa2.dat <- cbind(fa2.used,new_data)

# Plot FDG

> ggplot(data = fa2.dat,aes(x = FDG1,y = FDG2)) +

 geom_point(size = 0.05,aes(color = pseudotime)) +

 scale_color_viridis_c(name = 'Pseudotime',option = "C")+

 theme_classic() +

 xlab("FDG1") +

 ylab("FDG2")

87. **Visualize the expression of selected genes along the paths.** We plot erythroid lineage genes as the example.
  a. Add developmental stages information to the metadata.
    # Retrieve UMAP coordinates and annotate with cluster information
    > sc.clusters <-Human_HSPC@sc.clusters
    > umap.results <- Human_HSPC@umap.result
    > umap.results <- merge(umap.results,sc.clusters, by = "Cell")
    ### Add developmental stage information
    > umap.results$stage[umap.results$sampleID %in% c("1_eFL_1","1_eFL_2")]<- "eFL"
    > umap.results$stage[umap.results$sampleID %in% c("1_ABM_1")]<- "ABM"
    > umap.results$stage[umap.results$sampleID %in% c("1_FL_1","1_FL_2")]<- "FL"
    > umap.results$stage[umap.results$sampleID %in% c("1_FBM_1","1_FBM_2")]<- "FBM"
    > umap.results$stage[umap.results$sampleID %in% c("1_PBM_1","1_PBM_2")]<- "PBM"
  b. Define the path for the erythroid lineage based on the FDG, diffusion map, and Monocle3. We selected the path ‘cl1-cl7-cl12-cl3’ for the erythroid lineage.
    > Ery.path <- c("cl1","cl7","cl12","cl3")
  c. Extract cells from the erythroid trajectory for all stages.
    > umap.results.Ery <- umap.results[umap.results$louvain_cluster %in% Ery.path,]
    > cells.eFL <- umap.results.Ery$Cell[umap.results.Ery$stage == "eFL"]
    > cells.FL <- umap.results.Ery$Cell[umap.results.Ery$stage == "FL"]
    > cells.FBM <- umap.results.Ery$Cell[umap.results.Ery$stage == "FBM"]
    > cells.PBM <- umap.results.Ery$Cell[umap.results.Ery$stage == "PBM"]
    > cells.ABM <- umap.results.Ery$Cell[umap.results.Ery$stage == "ABM"]
  d. Extract genes known to be involved in the erythroid trajectory based on the pseudotime.
    > genes.E <- c("FAM178B","TRIB2","BLVRB","RHAG","PNMT",
      "APOE","APOC1","S100A6","KLF1","PVT1",
      "KCNH2","EPOR","MYH10","S100A4",
      "MYL4","EMP3","CYTOR","CNRIP1","GATA1","SMIM1")
    > matrix <- newcds@assays@data$counts
    > pt.matrix<-
     matrix[match(genes.E,rowData(newcds)[,1]),order(pseudotime(newcds))]
  e. Extract gene expression matrix for each group of cells.
    > pt.matrix.eFL <- ExtractMatrix(pt.matrix = pt.matrix,genes = genes.E,path =
     Ery.path,selected.cells = cells.eFL)
    > pt.matrix.FL <- ExtractMatrix(pt.matrix = pt.matrix,genes = genes.E,path =
     Ery.path,selected.cells = cells.FL)
    > pt.matrix.FBM <- ExtractMatrix(pt.matrix = pt.matrix,genes = genes.E,path =
     Ery.path,selected.cells = cells.FBM)
    > pt.matrix.PBM <- ExtractMatrix(pt.matrix = pt.matrix,genes = genes.E,path =
     Ery.path,selected.cells = cells.PBM)
    > pt.matrix.ABM <- ExtractMatrix(pt.matrix = pt.matrix,genes = genes.E,path =
     Ery.path,selected.cells = cells.ABM)
  f. Plot gene expression heatmap along the path of the different developmental stages (Figure 8H).
    > ht1 <- plot_development_heatmap(pt.matrix.eFL,subtitle = "eFL")
    > ht2 <- plot_development_heatmap(pt.matrix.FL,subtitle = "FL")
    > ht3 <- plot_development_heatmap(pt.matrix.FBM,subtitle = "FBM")
    > ht4 <- plot_development_heatmap(pt.matrix.PBM,subtitle = "PBM")
    > ht5 <- plot_development_heatmap(pt.matrix.ABM,subtitle = "ABM")
    > ht.full <- ht1+ht2+ht3+ht4+ht5
    > ht.full
  g. Extract gene expression from downsampled cells along the path from different developmental stages and pseudotime from ‘newcds’ object from step 83.
    > Ery.eFL <- ExtractCells(selected.cells = cells.eFL)
    > Ery.FL <- ExtractCells(selected.cells = cells.FL)
    > Ery.FBM <- ExtractCells(selected.cells = cells.FBM)
    > Ery.PBM <- ExtractCells(selected.cells = cells.PBM)
    > Ery.ABM <- ExtractCells(selected.cells = cells.ABM)
    > matrix <- newcds@assays@data$counts
    > matrix.total <-Matrix::colSums(matrix)
    > norm.matrix <-(t(t(matrix)/matrix.total))∗10000
    > expr.eFL <- norm.matrix[genes.E,Ery.eFL]
    > expr.eFL <- reshape2::melt(as.matrix(expr.eFL))
    > colnames(expr.eFL) <- c("Gene","Cell","NormUMI")
    > expr.eFL$Stage <- "eFL"
    > expr.FL <- norm.matrix[genes.E,Ery.FL]
    > expr.FL <- reshape2::melt(as.matrix(expr.FL))
    > colnames(expr.FL) <- c("Gene","Cell","NormUMI")
    > expr.FL$Stage <- "FL"
    > expr.FBM <- norm.matrix[genes.E,Ery.FBM]
    > expr.FBM <- reshape2::melt(as.matrix(expr.FBM))
    > colnames(expr.FBM) <- c("Gene","Cell","NormUMI")
    > expr.FBM$Stage <- "FBM"
    > expr.PaedBM <- norm.matrix[genes.E,Ery.PBM]
    > expr.PaedBM <- reshape2::melt(as.matrix(expr.PaedBM))
    > colnames(expr.PaedBM) <- c("Gene","Cell","NormUMI")
    > expr.PaedBM$Stage <- "PBM"
    > expr.AdultBM <- norm.matrix[genes.E,Ery.ABM]
    > expr.AdultBM <- reshape2::melt(as.matrix(expr.AdultBM))
    > colnames(expr.AdultBM) <- c("Gene","Cell","NormUMI")
    > expr.AdultBM$Stage <- "ABM"
    > expr.Ery <- rbind(expr.eFL,expr.FL,expr.FBM,expr.PaedBM,expr.AdultBM)
  h. Extract the pseudotime information from Monocle3 results.
    > pseudotime <- as.data.frame(pseudotime(newcds))
    > colnames(pseudotime) <- "pseudotime"
    > pseudotime$Cell <- rownames(pseudotime)
    > pseudotime$pseudotime[pseudotime$pseudotime %in% "Inf"] <- 0
    > pseudotime <- pseudotime[order(pseudotime$pseudotime,decreasing = F),]
  i. Merge gene expression data with pseudotime analysis results.
    > expr.Ery <- merge(expr.Ery,pseudotime,by = "Cell",)
  j. Visualize selected erythroid gene expression along the path (Figure 8I).
    > plot_genes(data = expr.Ery,genes = "GATA1")
    > plot_genes(data = expr.Ery,genes = "KLF1")

## Expected outcomes

The step-by-step protocols describe an analysis pipeline used in a recent publication (Roy et al., 2021). Here, we introduce SingCellaR as a tool to facilitate various data analyses and visualization of scRNA-seq data. We expect the outcomes from the pipelines can help reduce data analysis complications, speed up, and generate robust results. We summarize the expected outputs described in Table 1.

**Table 1:** The summary of expected outputs from the protocols

Key step	Step	Output definition	Output file; Figure
SingCellaR installation	1–3	Following these steps, a user should understand the installation processes of R packages. The protocols can help users successfully install SingCellaR and its dependencies on personal computers or high-performance workstation/computing clusters. We describe the possible issues for installing required Python modules in the R environment in the troubleshooting section. At the end of the step, we expect the user to run the command library(SingCellaR) successfully in the R terminal.	
Processing scRNA-seq for an individual sample	4–12	These steps are for the initial analysis of an individual sample. The output files consist of nine SingCellaR objects generated from each sample. These files are used for the downstream data integration. We show the process to select high-quality cells using multiple QC plots, and we perform data normalization and identification of highly variable genes. We expect the user to inspect the QC of cells when applied using distinct cut-off parameters.	eFL_1.SingCellaR.rdata, eFL_2.SingCellaR.rdata, FL_1.SingCellaR.rdata, FL_2.SingCellaR.rdata, FBM_1.SingCellaR.rdata, FBM_2.SingCellaR.rdata, PBM_1.SingCellaR.rdata, PBM_2.SingCellaR.rdata, ABM_1.SingCellaR.rdata; Figure 1
Integrating biological replicates	13–29	These steps describe the general procedures to integrate individual R objects derived from five developmental stages (eFL, FL, FBM, PBM, and ABM). The integration of two eFL samples from two donors is demonstrated. We show the SingCellaR’s functions to perform standard scRNA-seq analyses. All output files generated from these steps are available at Zenodo:https://doi.org/10.5281/zenodo.5879071.	eFL_All.SingCellaR.rdata, FL_All.SingCellaR.rdata, FBM_All.SingCellaR.rdata, PBM_All.SingCellaR.rdata, ABM_1.SingCellaR.rdata; Figure 2
Integrating samples from all tissue types	30–52	We show SingCellaR’s functionalities to support data integration from all samples. From these steps, the user should observe different results of applying distinct integrative methods implemented in SingCellaR. We demonstrate wrapper functions for Supervised Harmony, Harmony, Seurat and Scanorama integration, and Limma batch correction. We describe the benchmarking technique of different integrative results using AUCell analysis with LISI and kBET methods. The user should observe the objective measurement of integration from the plots to indicate the performance of each method for the integration of HSPC datasets.	supervised_harmony.UMAP.rds, harmony.UMAP.rds, Seurat_rpca.UMAP.rds, Scanorama.UMAP.rds, Limma.UMAP.rds, human_HSPC_rankings.AUCells.rdata, Human_HSPC.CellType_from_AUC_High.rds, Human_HSPC_All.SingCellaR.rdata; Figures 3, 4, and 5A
Cell type annotation	53–61	These steps describe how to use functions implemented in SingCellaR to annotate cell types. SingCellaR implements a GSEA-based approach using a comprehensive list of curated gene sets and visualization of marker genes to annotate cell types.	Human_HSPCs_preRankedGenes_for_GSEA.rdata Human_HSPC_marke_genes_per_cluster.txt; Figures 5B and 6
AUCell analysis	62–67	SingCellaR implements AUCell analysis to assign and define high-confident lineage-specific cells. We validate cell-type annotation and compare the differential abundance of different cell lineages across developmental stages using the wrapper function for DAseq analysis.	Human_HSPC_rankings.AUCells.rdata, human_HSPCs.AUCells.score.rdata, eFL_vs_FL.pdf; Figure 7
Trajectory analysis	68–87	We describe three distinct approaches, Force-directed graph, Diffusion map, and Monocle3, to infer cellular trajectories. We show how to transfer the results from SingCellaR to be analyzed in Monocle3.	Figure 8

The table shows a short description of protocol steps, output description, and expected output files and figures.

## Limitations

SingCellaR requires signature gene sets to perform the cell type annotation analysis. Thus, the user would have to compile and curate customized gene sets for the relevant system of interest. In this protocol, we provide 75 gene sets curated from previous studies relevant to hematopoiesis. SingCellaR still lacks 'automatic object transformation' to interact with other existing packages, such as Seurat. However, SingCellaR uses the SingleCellExperiment object, the standard object for storing single-cell experimental data in R. Therefore, the gene expression matrix and cell metadata can be extracted simply from the SingleCellExperiment object. This issue will be improved when SingCellaR is updated to the next version to support more interactions with other packages and ensure compatibility with relevant R packages incorporated in SingCellaR.

## Troubleshooting

### Problem 1

The user may encounter the following error when executing the function ‘runFA2_ForceDirectedGraph’:

Error in runFA2_ForceDirectedGraph(Human_HSPC,useIntegrativeEmbeddings = T, :

The fa2 python module is not installed!. Please install using pip (pip install fa2)

There are two potentially problems: the fa2 package is not installed; and the R environmental path for FA2 module in python is not found by R.

### Potential solution

FA2 installation.

The fa2 package can be installed as suggested in the SingCellaR installation section.

Conda environment is recommended by using ‘conda_create’ function after loading the reticulate package.

***Note:*** The fa2 package is not compatible with python 3.9 or higher versions.

python version configuration.

The user can use the following code to configure the python path:

### Set the python version into R environment.

use_python("/miniconda3/envs/r-reticulate/bin/python")

***Note:*** The user must change the python path as shown here to Conda-specific path found in the user’s computer.

### Open ‘∼/.Renviron’ in the terminal and add the following code:

RETICULATE_PATH="/miniconda3/envs/r-reticulate/bin/python"

### Restart R session and then use below code to check:

Sys.which("python")

"/miniconda3/envs/r-reticulate/bin/python" should be shown on the console.

The user can refer to Python version configuration tutorial in the reticulate R package found in this URL: https://rstudio.github.io/reticulate/articles/versions.html.

### Problem 2

The user may find this error when performing the runScanorama(Human_HSPC) function:

“Error in runScanorama(Human_HSPC) : The scanorama python module is not installed!. Please install using pip ('pip install scanorama')”

### Potential solution

The scanorama package can be installed as suggested in the SingCellaR installation section. The Python environment configuration can be found in Problem 1.

### Problem 3

UMAP/FDG plots may show different rotations from this protocol. This is caused by different software versions for generating plots and the seed number setting.

### Potential solution

This can be solved by setting a seed number (n.seed parameter) found in runUMAP and runFA2_ForceDirectedGraph functions.

### Problem 4

The user may encounter running time and memory issues when performing the AUCell_buildRankings function for a large-scale dataset.

### Potential solution

The user can use the alternative function named ‘Build_AUCell_Rankings_Fast’ to speed up running time and use less memory for ranking gene expression for each cell.

### Problem 5

The kBET score is used to benchmark the integration results from different integrative methods. The user may encounter slightly different kBET scores from this protocol. This is due to the different seed number settings and the number of subsampling cells for kBET analysis. More running time and memory will be used if the user sets the high number of the downsample size in kBET analysis.

### Potential solution

This issue can be solved by setting the seed number prior to running kBET using the ‘set.seed’ function. The user can subsample and fix the number of cells for the kBET analysis using the n.sample parameter described in the ‘runKBET’ function.

## Resource availability

### Lead contact

Further information and requests for resources and reagents should be directed to and will be fulfilled by the lead contact, Supat Thongjuea (supat.thongjuea@imm.ox.ac.uk).

### Materials availability

This study did not generate new unique reagents.

## Data Availability

• SingCellaR open-source codes, cellranger pipeline outputs, R codes and pre-processed R objects for this protocol are available and maintained on GitHub and Zenodo listed in the key resources table. • Any additional information required to reanalyze the data reported in this paper is available from the lead contact upon request.
